# Age‐Associated Four‐Gene Prognostic Signature in Breast Cancer

**DOI:** 10.1002/cnr2.70670

**Published:** 2026-09-01

**Authors:** Shiro Uchida

**Affiliations:** ^1^ Division of Diagnostic Pathology Kikuna Memorial Hospital Yokohama Japan; ^2^ Department of Human Pathology Juntendo University School of Medicine Tokyo Japan; ^3^ Division of Pathology Shizuoka Cancer Center Shizuoka Japan

**Keywords:** breast cancer, disease‐free survival, the cancer genome atlas, young‐onset

## Abstract

**Background:**

Young‐onset breast cancer is associated with inferior disease‐free survival (DFS), but the contribution of additional molecular heterogeneity remains unclear.

**Aims:**

To identify an exploratory age‐associated gene expression signature linked to recurrence‐related outcomes and evaluate its prognostic association.

**Methods and Results:**

We analyzed clinicopathological and RNA‐sequencing data from 821 patients with Stages I–III invasive ductal or lobular carcinoma in The Cancer Genome Atlas, including 142 patients aged ≤ 45 years. Genes associated with both age and DFS were screened, followed by LASSO‐Cox and stepwise multivariable Cox regression. A four‐gene signature (Sig4: C4orf14 [NOA1], LINC01124, ZNF704, and AGFG2) was identified. Young patients had significantly worse DFS than older patients, whereas overall and disease‐specific survival did not differ significantly. After adjustment for clinicopathological factors, young age remained associated with worse DFS. Following inclusion of the continuous Sig4 score, the age association was attenuated and no longer statistically significant, while Sig4 remained independently associated with worse DFS. Sig4‐high tumors were enriched for proliferation, cell‐cycle, DNA‐repair, metabolic, and stress‐response pathways. In METABRIC, the fixed TCGA‐derived Sig4 score was associated with worse relapse‐free survival in the overall cohort but not in patients aged ≤ 45 years.

**Conclusion:**

Sig4 is an exploratory age‐associated four‐gene signature with potential general prognostic relevance in breast cancer. Its utility for risk stratification specifically in young‐onset breast cancer was not externally validated and requires confirmation in independent prospective cohorts enriched for young patients.

## 1 Introduction

Young age at diagnosis is widely recognized as a poor prognostic factor in breast cancer, the fourth leading cause of cancer‐related death worldwide [1]. However, this disadvantage tends to primarily manifest as an increased risk of recurrence and decreased disease‐free survival (DFS), rather than as differences in overall survival (OS) or disease‐specific survival (DSS) [2, 3, 4]. The independent prognostic significance of young age remains a topic of debate, with early epidemiological studies suggesting that it merely reflects confounding by advanced‐stage disease and unfavorable tumor biology (e.g., subtype and receptor expression) rather than age itself [5]. Advocates of the confounding hypothesis argue that the apparently diminished survival among young patients can be explained by adverse pathological features, such as high‐grade tumor, lymphovascular invasion, and elevated proliferative activity, with age losing its significance in multivariable analyses [6]. Supporting this perspective, grade and Ki‐67 have been reported as the principal determinants of outcome rather than age itself, even in very young cohorts [7]. From a staging perspective, young patients are more frequently diagnosed at advanced stages, which may largely account for their inferior outcomes [8]. At the molecular level, young‐onset breast cancers are enriched in features associated with a poor prognosis, including low estrogen receptor/progesterone receptor (ER/PR) expression, high human epidermal growth factor receptor 2 (HER2) and epidermal growth factor receptor expressions, and a high prevalence of the basal‐like subtype. These features suggest that the skewed subtype distribution may account for their adverse outcomes [2]. Conversely, several studies have demonstrated that young age remains a poor prognostic factor even after adjustment for stage and receptor status [9]. A large‐scale analysis demonstrated increased mortality among young patients even in early‐stage disease, suggesting that the disadvantage cannot be attributed solely to advanced presentation [10]. Furthermore, patients younger than 35 years of age demonstrate increased risks of recurrence and distant metastasis that are not fully explained by pathological factors [11]. This age‐associated effect may also be context‐dependent, with a study reporting that young age confers a disadvantage only in low‐to‐intermediate‐risk groups [4]. Subtype‐specific analyses have further revealed that age serves as a prognostic factor for luminal A tumors, indicating that subtype adjustment does not eliminate the age effect [12]. Thus, it remains unclear whether the poor prognosis of young‐onset breast cancer can be explained by stage and subtype or reflects additional biological mechanisms [3].

We therefore aimed to identify a gene expression‐based molecular signature associated with both young age and adverse DFS in breast cancer, beyond conventional clinicopathological factors. We further examined whether this signature provided additional prognostic information in young‐onset breast cancer.

## 2 Methods

The study used publicly available and deidentified data from The Cancer Genome Atlas (TCGA) and cBioPortal; therefore, ethics approval and consent to participate were not applicable.

### 2.1 Study Population Assembly and Age‐Based Stratification of Invasive Ductal Carcinoma and Invasive Lobular Carcinoma

Genomic and clinical data were obtained from the TCGA PanCancer Atlas Breast Invasive Carcinoma cohort via cBioPortal (*n* = 1084) [13]. Cases annotated in the “Cancer Type Detailed” field as invasive ductal carcinoma (IDC; *n* = 780) or invasive lobular carcinoma (ILC; *n* = 201) were initially selected, yielding 981 cases. Among these, six had discordant annotations indicating other histological types, including medullary carcinoma, and were excluded, leaving 975 cases. After excluding 35 patients with Stage IV disease, unknown pathological stage, or M1 status, 940 patients remained. A further 119 patients with a Normal‐like or missing PAM50 subtype were excluded, although the biological interpretation of the Normal‐like subtype remains debated [14]. The final analytical cohort comprised 821 patients, including 142 patients aged ≤ 45 years and 679 patients aged > 45 years.

**FIGURE 1:**
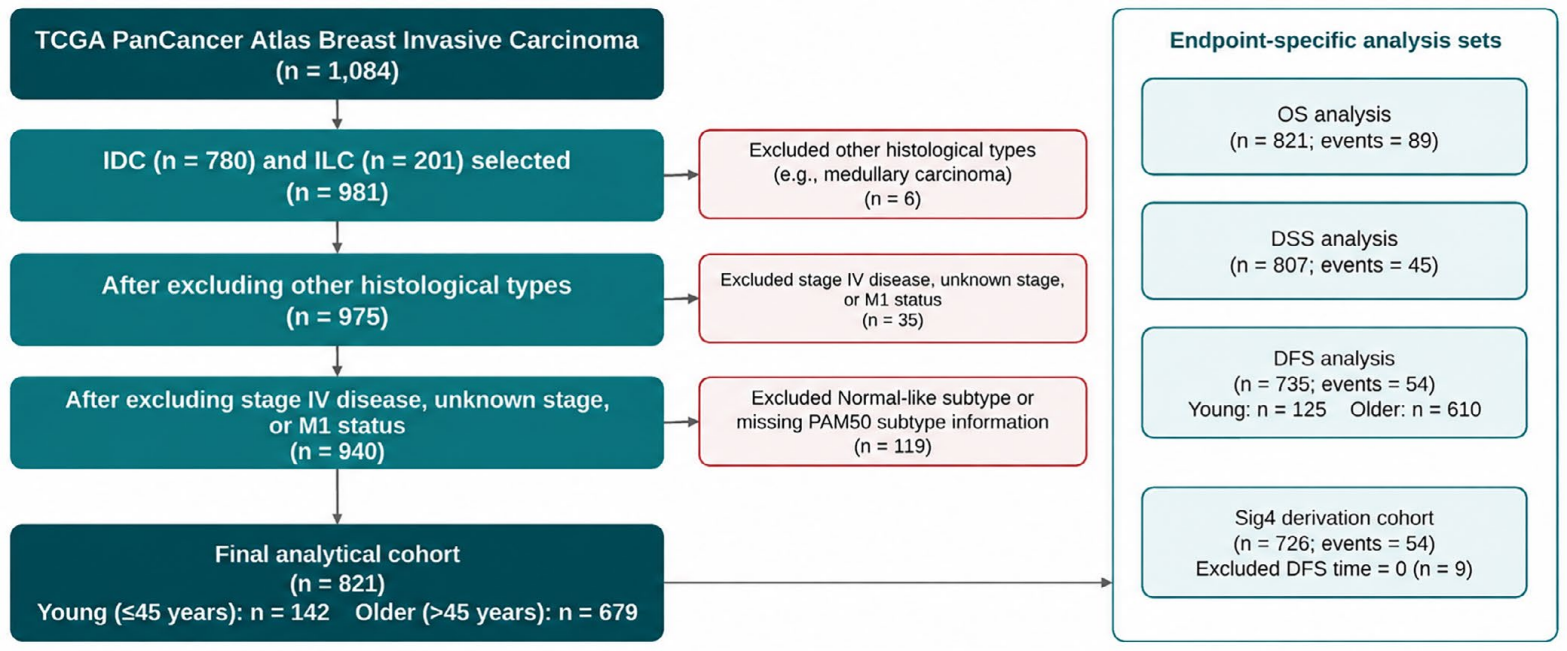
Flowchart of study cohort selection from the TCGA PanCancer Atlas Breast Invasive Carcinoma dataset. Patients with invasive ductal carcinoma or invasive lobular carcinoma were selected from the TCGA cohort. After applying the exclusion criteria, including stage IV disease, M1 status, unknown stage, normal‐like subtype, or missing PAM50 data, 821 patients were retained. ER, estrogen receptor; HER2, human epidermal growth factor receptor 2; PR, progesterone receptor; TCGA, The Cancer Genome Atlas.

For clinicopathological classification, the receptor status for ER, PR, and HER2 was annotated using the TCGA Firehose clinical repository, only for cases corresponding to these 821 patients (Figure 1). No additional Firehose‐only cases were included. For cases without matching Firehose receptor information, the receptor status for ER, PR, and HER2 was recorded as missing. The receptor status in Firehose is primarily derived from immunohistochemical (IHC) assessment. For HER2, equivocal IHC results were resolved using fluorescence in situ hybridization (FISH) results when available, and the final status was determined based on the integrated IHC/FISH assessment. Although the definition of “young” breast cancer remains unsettled, an age cutoff of 45 years was adopted in this study, consistent with previous genomic and transcriptomic studies [15, 16]. Patients were classified into a young group (≤ 45 years) and an older group (> 45 years). To address potential concerns regarding the arbitrariness of this threshold, sensitivity analyses were performed using alternative age cutoffs of 35, 40, and 50 years.

### 2.2 Comparative Analyses of Clinicopathological Features

Clinicopathological characteristics, including ER, PR, HER2, subtype, histological type, T factor, N factor, and pathological stage, were compared between the young and older groups. Missing data labeled as “missing” were excluded from percentage calculations, and statistical comparisons were then performed between the two age groups. T category, pathological stage, PAM50 subtype, and histological type were available for all included patients, whereas N category was nonevaluable (NX) in seven patients (0.9%). Missing receptor‐status data were excluded from the descriptive percentages and group comparisons.

### 2.3 RNA Sequencing (RNA‐seq) Data Acquisition and Preprocessing

RNA‐seq gene expression data (“RNA Seq V2 RNA‐Seq by Expectation‐Maximization [RSEM]”) were obtained from the TCGA PanCancer Atlas Breast Invasive Carcinoma cohort via cBioPortal. Analyses were performed at the gene level. Expression values were log_2_‐transformed as log_2_(RSEM + 1) before downstream analyses. Gene expression data were matched with clinical data by patient identifiers. Among the 821 patients in the final analytical cohort, 735 had available DFS time and event information. To derive the signature, nine patients with a recorded DFS time of 0 were excluded, yielding a derivation cohort of 726 patients.

### 2.4 Construction of the Sig4 Gene Expression Signature

To construct the Sig4 signature, genes associated with both age and DFS were first identified in the TCGA derivation cohort. Age‐associated genes were identified by comparing differential gene expression between young and older patients. *p*‐Values were adjusted for multiple testing using the Benjamini–Hochberg false discovery rate (FDR) method, and genes with an FDR < 0.1 were defined as age‐associated differentially expressed genes. To identify candidate DFS‐associated genes, a univariable Cox proportional hazards regression analysis was performed for each gene using DFS as the endpoint. Given the limited number of DFS events, a prespecified *p*‐value threshold of < 0.01 served as a screening criterion. Genes meeting both criteria—age‐associated differential expression (FDR < 0.1) and candidate association with DFS in univariable analysis (*p* < 0.01)—were selected by intersecting the two gene sets, yielding 11 candidate genes. These 11 genes were subsequently entered into a least absolute shrinkage and selection operator (LASSO)‐Cox regression model. The penalty parameter was optimized by 10‐fold cross‐validation, and the model at lambda.min was selected, retaining eight genes.

To obtain a more parsimonious model, these genes were further reduced using stepwise multivariable Cox proportional hazards regression based on the Akaike information criterion (AIC). This process yielded a final four‐gene model consisting of C4orf14 (*NOA1*), *LINC01124*, *ZNF704*, and *AGFG2*. Thus, inclusion of a gene in the final Sig4 model was determined by the penalized and multivariable model‐selection procedure rather than by its individual *p*‐value in the final multivariable model. A multivariable Cox proportional hazards model including these four genes was then fitted in the derivation cohort. The resulting regression coefficients were used to define the Sig4 score. For each patient, the Sig4 score was calculated as a weighted linear combination of the log_2_‐transformed expression values of the four selected genes:

Sig4 score=Σβᵢ×Gᵢ,

where *βᵢ* represents the Cox regression coefficient for gene *i* derived from the final multivariable Cox model, and *Gᵢ* denotes the log_2_(RSEM + 1)‐transformed expression value of gene *i*. The fixed regression coefficients used to calculate the Sig4 score are provided in Supporting Information 1. The regression coefficients estimated in the derivation cohort were subsequently applied to the full matched cohort to calculate the Sig4 score for each case. For downstream analyses, the Sig4 score was primarily modeled as a continuous variable per 1‐standard deviation (SD) increase in Cox regression analyses. To visualize survival differences, the score was also dichotomized at its median of 0.15841 in the TCGA cohort into Sig4‐high and Sig4‐low groups.

### 2.5 Survival Analysis and Cox Regression Analyses

OS, DSS, and DFS were analyzed using the survival endpoints provided by TCGA via cBioPortal. As survival time or event indicators were missing for some cases, the number of included patients differed across endpoints (OS, *n* = 821; DSS, *n* = 807; DFS, *n* = 735); the corresponding numbers of events were 89 for OS, 45 for DSS, and 54 for DFS. DFS was used as the primary endpoint for prognostic modeling. Cox proportional hazards regression analyses were performed to evaluate the associations of clinicopathological variables and the Sig4 score with DFS. The Sig4 score was primarily modeled as a continuous variable per 1‐SD increase. Sequential multivariable models were constructed to assess whether the incorporation of the Sig4 score attenuated the prognostic effect of young age and provided incremental prognostic information beyond established clinicopathological factors. In the primary clinicopathological multivariable models, PAM50 subtype was entered as separate categorical variables, with Luminal A used as the reference category and Luminal B, HER2‐enriched, and Basal‐like tumors modeled individually. To illustrate how the estimated association with young age changed as covariates were added, a parsimonious series of models used a binary luminal versus nonluminal subtype variable. This simplified specification was used only for the sequential models and was not the only subtype adjustment performed. Given the limited number of DFS events, additional correlated clinicopathological variables, including T and N categories and receptor status, were not simultaneously entered into the primary models to avoid overparameterization and unstable coefficient estimates. Pathological stage was used as a composite measure of the anatomical extent of disease.

To evaluate the prognostic impact of the Sig4 score in young patients, DFS was further analyzed after stratifying the young cohort by Sig4 status. Young patients were categorized into Sig4‐high and Sig4‐low groups with the median Sig4 score of 0.15841 derived from the overall TCGA cohort, rather than recalculating a separate median within the young subgroup. Cox regression analyses were also performed for the young subgroup, including pathological stage, intrinsic subtype, histology, and the Sig4 score as covariates. Because of the limited number of DFS events in the young‐subgroup model, the stability of the estimated association with the continuous Sig4 score was evaluated with nonparametric bootstrap resampling with 1000 iterations. Bootstrap percentile confidence intervals (CIs) were calculated from the resampled Cox regression estimates. The continuous Sig4 score per 1‐SD increase was treated as the primary analysis, whereas median‐based dichotomization was used only for descriptive Kaplan–Meier visualization. The proportional hazards assumption was formally assessed for all reported Cox proportional hazards models with scaled Schoenfeld residual‐based tests. Both covariate‐specific and global tests were evaluated. When evidence of non‐proportionality was identified, scaled Schoenfeld residual plots were visually inspected and additional sensitivity analyses were performed.

### 2.6 Gene Set Enrichment Analysis (GSEA) and Single‐Sample GSEA (ssGSEA)

GSEA was performed using curated gene sets from the Molecular Signatures Database (MSigDB) to explore biological pathways associated with the Sig4 signature. Patients were stratified into Sig4‐high and Sig4‐low groups with the same overall TCGA median Sig4 cutoff of 0.15841. Enrichment significance was assessed using the FDR *q*‐value, and gene sets with FDR *q* < 0.25 were considered significant, in accordance with the original GSEA framework and recommended practice in GSEA documentation. MSigDB gene sets, including the Hallmark collection, were obtained from the MSigDB. To further evaluate pathway activity at the individual‐sample level, ssGSEA was performed for selected Hallmark pathways, and ssGSEA scores were compared between Sig4‐high and Sig4‐low tumors. Differences in ssGSEA scores between groups were assessed using the Mann–Whitney *U* test.

### 2.7 External Validation of the Sig4 Signature in the METABRIC Cohort

To externally validate the Sig4 signature derived from the TCGA cohort, we applied the fixed TCGA‐derived Sig4 model to an independent METABRIC cohort. Cases were selected using exclusion criteria designed to be conceptually consistent with those used in the TCGA analytical cohort, and matched clinical and gene expression data were analyzed. Gene expression data were obtained from the Illumina HT‐12 v3 microarray platform. The expression matrix contained Hugo_Symbol and Entrez_Gene_Id, with sample‐level expression values arranged in columns. Clinical and expression data were matched using Sample ID. The current Sig4 signature consisted of four genes: C4orf14, LINC01124, ZNF704, and AGFG2. In the METABRIC expression dataset, C4orf14 was annotated as NOA1; therefore, NOA1 was used as the corresponding gene for C4orf14 in the external validation analysis. Because some gene symbols were represented by multiple rows in the microarray dataset, expression values were collapsed to the gene level by averaging duplicated rows for each Hugo_Symbol. Expression values for the four Sig4 genes were then extracted and standardized across the METABRIC cohort using *z*‐score transformation. The Sig4 score was calculated as a weighted sum of standardized gene expression values using the fixed Cox regression coefficients derived from the TCGA cohort. The score was defined as follows:

Sig4 score = 0.685 × *z*(NOA1/C4orf14) − 0.116 × *z*(LINC01124) − 0.237 × *z*(ZNF704) − 0.471 × *z*(AGFG2).

The coefficients were not re‐estimated in METABRIC. Patients were dichotomized into Sig4‐high and Sig4‐low groups using the median Sig4 score in the METABRIC validation cohort. Relapse‐free survival (RFS) was used as the validation endpoint. This endpoint was chosen because DFS, as defined in TCGA, was not available in METABRIC. Although DFS and RFS both capture recurrence‐related outcomes, their database‐specific definitions and event components are not necessarily identical. Therefore, the METABRIC analysis was considered an external validation of the prognostic association of Sig4 with a related recurrence endpoint rather than an exact replication of the TCGA DFS analysis. Kaplan–Meier curves were generated and compared using the log‐rank test. Univariable and multivariable Cox proportional hazards regression analyses were also performed to evaluate the association of young age and Sig4 status with RFS. The multivariable model included age group, Sig4 group, Stages III versus I–II disease, luminal versus nonluminal subtype, and histology (ILC vs. IDC). Following formal assessment of the proportional hazards assumption, an additional stratified Cox sensitivity model was fitted with stage and luminal versus non‐luminal subtype as stratification factors, thereby allowing separate baseline hazards across strata. Because residual nonproportionality remained for young age, a further sensitivity model incorporating a time‐varying coefficient for young age, defined as an interaction with log(time + 1), was fitted. As a supplementary analysis, we also examined whether the TCGA‐derived Sig4 score could further stratify RFS among young patients aged ≤ 45 years within METABRIC. For this analysis, Sig4 was evaluated as a continuous variable per 1‐SD increase.

### 2.8 Use of Artificial Intelligence Tools

ChatGPT, a large language model developed by OpenAI, was used to assist with language editing, grammar and spelling checks, improvement of clarity, preparation and troubleshooting of Python code for statistical analysis and data visualization, and verification of consistency between the manuscript text, figures, and tables. ChatGPT was not used to generate original research data, alter or manipulate research results, determine the study design, or make scientific conclusions. All analyses, numerical results, figures, tables, interpretations, and manuscript content were critically reviewed, verified, and approved by the author, who takes full responsibility for the integrity and accuracy of the submitted work.

### 2.9 Statistical Analysis

Clinicopathological characteristics and subtype distributions between the young and older cohorts were compared using the *χ* ^2^ test. Missing data labeled as “Missing” were excluded from percentage calculations. Survival curves were estimated using the Kaplan–Meier method and compared using the log‐rank test. To quantify absolute differences in DFS between age groups, the 5‐ and 10‐year DFS rates with 95% CIs, DFS event rates, and restricted mean survival time (RMST) at 5 and 10 years were additionally calculated. Cox proportional hazards regression analyses were used to estimate hazard ratios (HRs) and 95% CIs for DFS. Univariate analyses were performed for individual clinicopathological variables, and multivariable models were constructed as described above.

The Sig4 score was derived in R version 4.5.2 (R Foundation for Statistical Computing, Vienna, Austria), with feature selection thresholds predefined during signature construction, as described above. Proportional hazards testing and the corresponding stratified and time‐varying coefficient sensitivity analyses were also conducted in R version 4.5.2 with the survival package. All other statistical analyses were performed in Python version 3.13 (Python Software Foundation, Wilmington, DE, USA). All tests were two‐sided. Statistical significance was defined as *p* < 0.05 for the primary statistical analyses, unless otherwise specified. All analytical cutoff values were prespecified or defined as follows. Young age was primarily defined as ≤ 45 years, with alternative thresholds of ≤ 35, ≤ 40, and ≤ 50 years examined in sensitivity analyses. Age‐associated differentially expressed genes were defined with an FDR threshold of < 0.10, and candidate DFS‐associated genes were screened with a univariable Cox regression threshold of *p* < 0.01. The LASSO‐Cox model was selected at lambda.min determined by 10‐fold cross‐validation. In TCGA, Sig4‐high and Sig4‐low groups were defined with the median Sig4 score of 0.15841. For the METABRIC validation cohort, the corresponding cohort‐specific median cutoff was 0.03817. Hallmark gene sets with an FDR *q*‐value < 0.25 were considered significantly enriched.

## 3 Results

### 3.1 Comparison of Clinicopathological Features Between Young and Older Patients

A total of 821 patients were included, comprising 142 young and 679 older patients. No significant differences were observed in ER status, PR status, HER2 status, intrinsic subtype, T factor, or overall pathological stage distribution between the two groups. In contrast, histological type differed significantly according to age, with IDC being more frequent in young patients than in older patients (94.4% vs. 79.4%, *p* < 0.001). Moreover, the N factor differed significantly between the groups (*p* = 0.024), with young patients exhibiting a lower proportion of N0 disease (38.0% vs. 51.2%) and a higher proportion of N1 disease (43.7% vs. 32.0%) than older patients (Table 1). Overall, young patients were enriched for IDC histology and nodal involvement. Clinical data were generally complete for the variables used in the principal multivariable models. Pathological stage, T category, PAM50 subtype, and histology were available for all 821 patients, whereas N category was recorded as NX in 7 patients (0.9%). ER status was missing in 58 patients (7.1%), PR status was missing or indeterminate in 61 patients (7.4%), and integrated HER2 status was missing or indeterminate in 103 patients (12.5%). The proportions of missing receptor‐status data were slightly higher in young than in older patients, but the absolute differences were modest. DFS time or event information was unavailable for 86 patients (10.5%), including 17 of 142 young patients (12.0%) and 69 of 679 older patients (10.2%).

**TABLE 1:** Clinicopathological features of the analyzed cohort (*n* = 821).

Category		Young group (*n* = 142)	Older group (*n* = 679)	*p*
ER (%)	Positive	96 (74.4)	507 (80.0)	0.196
	Negative	33 (25.6)	127 (20.0)	
	Missing	13	45	
PR (%)	Positive	86 (66.7)	438 (69.4)	0.610
	Negative	43 (33.3)	193 (30.6)	
	Missing	13	48	
HER2 (%)	Positive	26 (21.3)	113 (19.0)	0.636
	Negative	96 (78.7)	483 (81.0)	
	Missing	20	83	
Subtype (%)	Luminal A	67 (47.2)	367 (54.1)	0.316
	Luminal B	31 (21.8)	142 (20.9)	
	HER2	12 (8.5)	58 (8.5)	
	Basal‐like	32 (22.5)	112 (16.5)	
Histology (%)	IDC	134 (94.4)	539 (79.4)	< 0.001
	ILC	8 (5.6)	140 (20.6)	
T category (T) (%)	T1	32 (22.5)	187 (27.5)	0.535
	T2	88 (62.0)	408 (60.1)	
	T3	18 (12.7)	66 (9.7)	
	T4	4 (2.8)	18 (2.7)	
N category (N) (%)	N0	54 (38.0)	344 (51.2)	0.024
	N1	62 (43.7)	215 (32.0)	
	N2	16 (11.3)	77 (11.5)	
	N3	10 (7.0)	36 (5.4)	
Stage (%)	I	20 (14.1)	126 (18.6)	0.337
	II	86 (60.6)	408 (60.1)	
	III	36 (25.4)	145 (21.4)	

Abbreviations: ER, estrogen receptor; HER2, human epidermal growth factor receptor 2; IDC, invasive ductal carcinoma; ILC, invasive lobular carcinoma; N/A, not applicable; PR, progesterone receptor.

### 3.2 Kaplan–Meier Analysis of OS, DSS, and DFS According to Age

**FIGURE 2:**
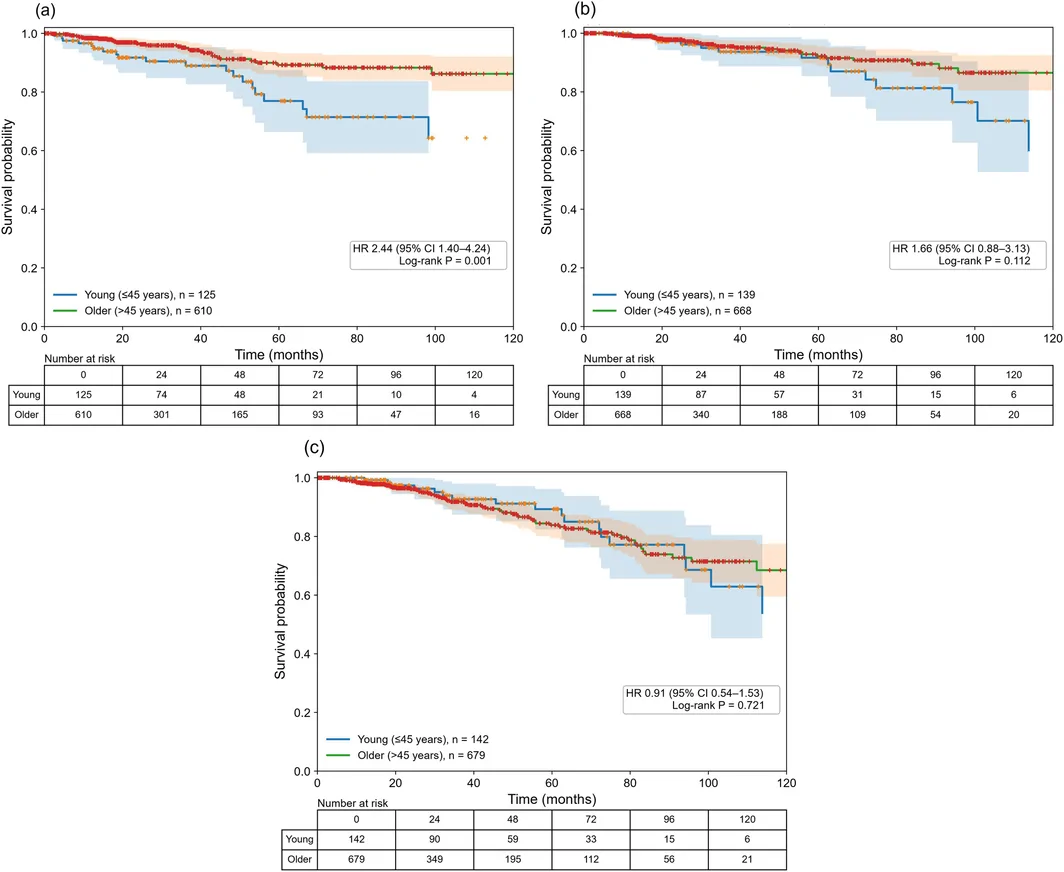
Kaplan–Meier analysis of OS, DSS, and DFS according to age. (a) DFS according to the age group (≤ 45 vs. > 45 years). Kaplan–Meier curves for DFS in young and older patients in the TCGA cohort. Survival differences were assessed using the log‐rank test. (b) DSS according to the age group (≤ 45 vs. > 45 years). Kaplan–Meier curves for DSS in young and older patients in the TCGA cohort. Survival differences were assessed using the log‐rank test. (c) OS according to the age group (≤ 45 vs. > 45 years). Kaplan–Meier curves for OS in young and older patients in the TCGA cohort. Survival differences were assessed using the log‐rank test. Kaplan–Meier curves include 95% confidence bands, censoring marks, and numbers at risk. Hazard ratios and 95% confidence intervals were estimated with univariable Cox proportional hazards regression, and *p*‐values were obtained with the log‐rank test. DFS, disease‐free survival; DSS, disease‐specific survival; OS, overall survival; TCGA, The Cancer Genome Atlas.

Survival analyses included 821 patients for OS, 807 patients for DSS, and 735 patients for DFS. The OS cohort comprised 142 young and 679 older patients, with 89 deaths overall. The DSS cohort comprised 139 young and 668 older patients, with 45 disease‐specific deaths. The DFS cohort comprised 125 young and 610 older patients, with 54 DFS events, including 20 in young patients and 34 in older patients. Median follow‐up estimated with the reverse Kaplan–Meier method was 26.7 months for DFS overall, 38.8 months in young patients, and 24.8 months in older patients. Young patients demonstrated significantly worse DFS than older patients (*p* = 0.001), whereas OS and DSS did not differ significantly between the two groups (OS, *p* = 0.721; DSS, *p* = 0.112) (Figure 2a–c).

### 3.3 DFS Event Rates and RMST

DFS event rates and RMST were evaluated to quantify the magnitude and timing of survival differences between age groups (Table 2). The estimated 5‐year DFS event rate (1 − Kaplan–Meier estimate) was 23.1% in young patients and 10.8% in older patients, increasing to 35.7% and 13.8% at 10 years, respectively. RMST analysis identified a significant difference at 5 years (54.2 vs. 57.1 months; Δ = −2.9 months, *p* = 0.025), with this difference becoming more pronounced at 10 years (95.9 vs. 109.8 months; Δ = −13.9 months, *p* = 0.003).

**TABLE 2:** Quantitative summary of DFS, recurrence rate, and RMST by age group.

Metric	Young group	Older group	Δ (young–older)	*p*
DFS (%) at 5 years	76.9	89.2	−12.3	
DFS (%) at 10 years	64.3	86.2	−21.9	
DFS event rate (%) at 5 years	23.1	10.8	12.3	
DFS event rate (%) at 10 years	35.7	13.8	21.9	
RMST (months) up to 5 years	54.2	57.1	−2.9	0.025
RMST (months) up to 10 years	95.9	109.8	−13.9	0.003

Abbreviations: DFS, disease‐free survival; RMST, restricted mean survival time.

### 3.4 Identification of a Four‐Gene Signature Associated With Young Age and a Poor Prognosis

Using the TCGA derivation cohort of 726 patients with matched RNA‐seq and DFS data, differential expression analysis identified 614 age‐associated genes (FDR < 0.1). Univariable Cox proportional hazards regression analysis identified 529 prognostic genes associated with DFS (*p* < 0.01). These two gene sets were overlapped, yielding 11 candidate genes, which were incorporated into the LASSO‐Cox regression model. Genes retained at lambda.min were further reduced using stepwise multivariable Cox regression based on AIC, resulting in a final four‐gene signature consisting of C4orf14 (*NOA1*), *LINC01124*, *ZNF704*, and *AGFG2*, and designated as Sig4.

**FIGURE 3:**
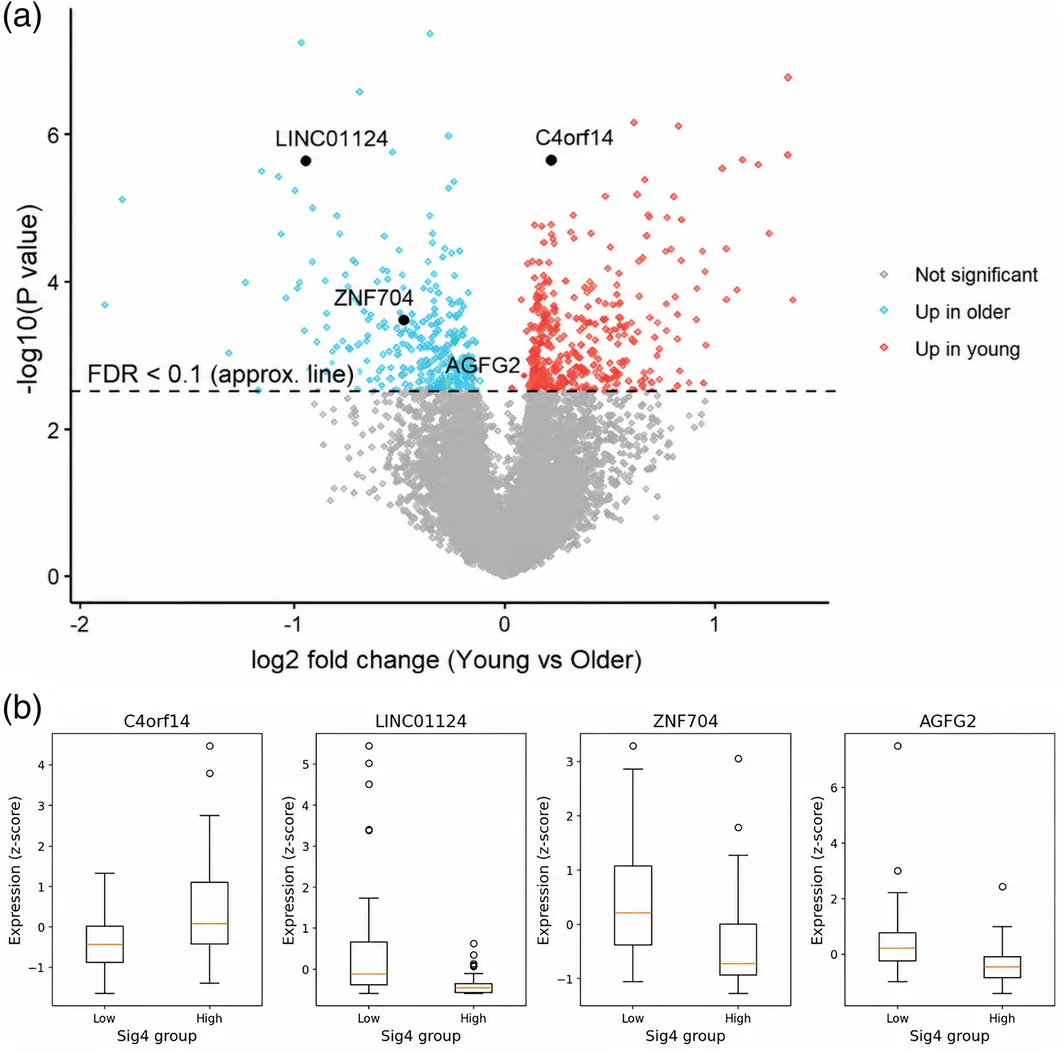
Identification of a four‐gene signature associated with young age and a poor prognosis. (a) Volcano plot of age‐associated differential gene expression highlighting Sig4 component genes. Volcano plot depicting differential gene expression between young and older patients in The Cancer Genome Atlas cohort. The *x*‐axis indicates log_2_ fold change, and the *y*‐axis indicates the ‐log_10_ FDR‐adjusted *p*‐value. The four genes comprising Sig4 (*C4orf14 [NOA1]*, *LINC01124*, *ZNF704*, and *AGFG2*) are highlighted. (b) Expression of Sig4 component genes in young patients. Boxplots depicting the expression of the four Sig4 component genes in young patients stratified by Sig4 status (high vs. low). Center lines indicate medians, boxes indicate IQRs, and whiskers extend to 1.5 × IQR. FDR, false discovery rate; IQR, interquartile range.

In the volcano plot, the four Sig4 genes were distributed across distinct regions rather than forming a single clustered signal, suggesting that the signature was not driven by a single dominant transcriptional change (Figure 3a). Consistently, boxplot analyses for young patients revealed distinct expression patterns between the Sig4‐high and Sig4‐low groups across all four constituent genes (Figure 3b), supporting the interpretation that the Sig4 score reflects the combined contribution of multiple genes.

### 3.5 Prognostic Determinants of DFS in Young Patients

**FIGURE 4:**
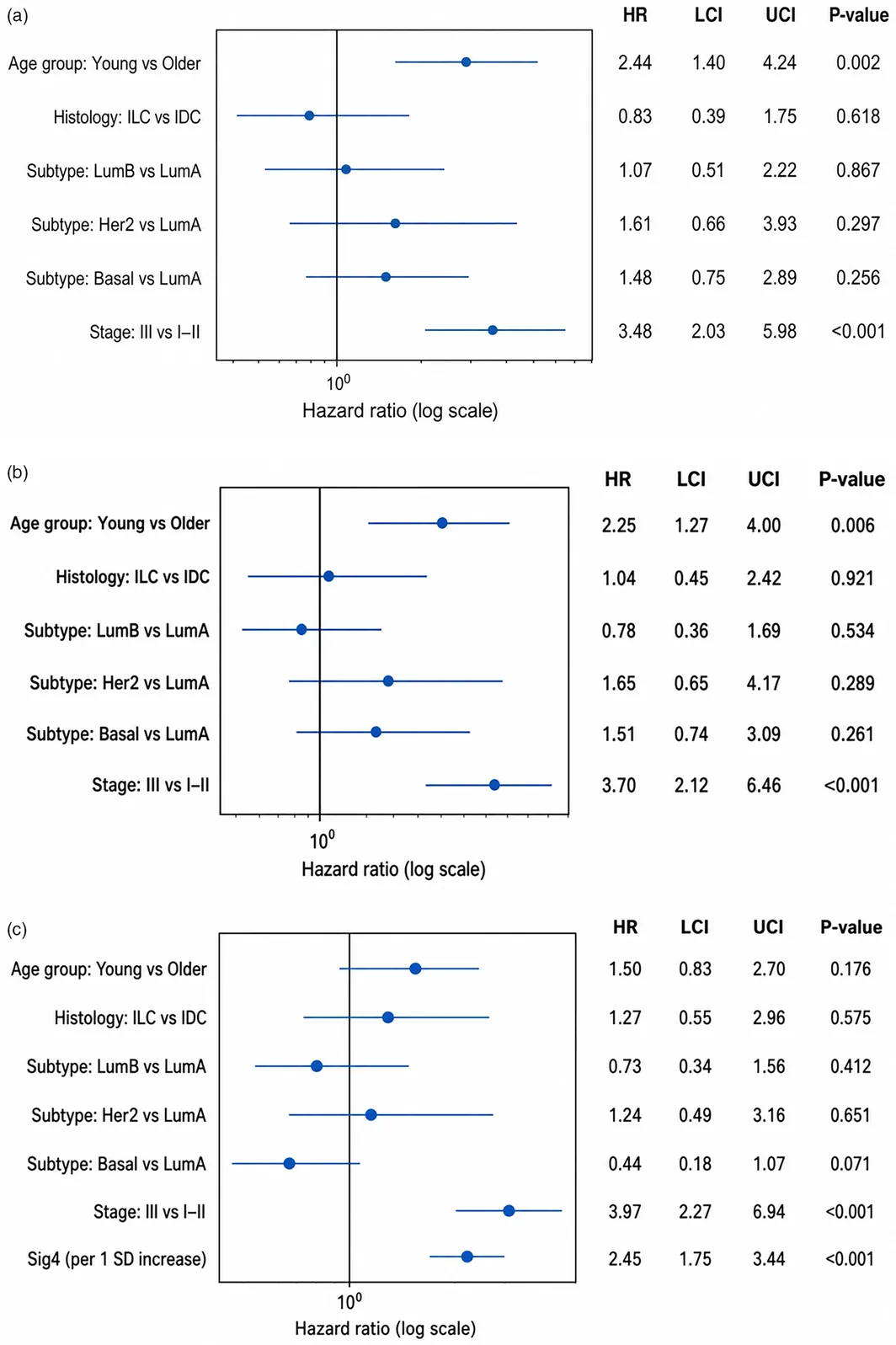
Prognostic determinants of DFS in young patients. (a) Univariable Cox proportional hazards analysis for DFS. Forest plot depicting HRs with 95% CIs from univariable Cox proportional hazards models for DFS in the TCGA cohort. Age group, histology, intrinsic subtype, and pathological stage were analyzed. Lower and upper CIs are presented as LCI and UCI, respectively. (b) Multivariable Cox proportional hazards analysis for DFS. Forest plot depicting HRs with 95% CIs from a multivariable Cox proportional hazards model for DFS in the TCGA cohort, incorporating age group, histology, intrinsic subtype, and pathological stage. Lower and upper CIs are presented as LCI and UCI, respectively. (c) Multivariable Cox proportional hazards analysis for DFS, including the continuous Sig4 score. Forest plot depicting HRs with 95% CIs from a multivariable Cox proportional hazards model for DFS in the TCGA cohort, incorporating age group, histology, intrinsic subtype, pathological stage, and the Sig4 score modeled as a continuous variable per 1‐SD increase. Lower and upper CIs are presented as LCI and UCI, respectively. CI, confidence interval; DFS, disease‐free survival; HER2, human epidermal growth factor receptor 2; HR, hazard ratio; IDC, invasive ductal carcinoma; ILC, invasive lobular carcinoma; Lum A, Luminal A; Lum B, Luminal B; TCGA, The Cancer Genome Atlas.

Cox proportional hazards regression was performed to identify factors underlying the unfavorable DFS observed in young patients. In univariable analyses (Figure 4a, Supporting Information 2), young age was associated with a poor DFS (HR 2.44, 95% CI: 1.40–4.24; *p* = 0.002), as was Stage III disease (HR 3.48, 95% CI: 2.03–5.98; *p* < 0.001). Histological type and intrinsic subtype were not significantly associated with DFS in univariable analyses. In multivariable models adjusted for histology, pathological stage, and PAM50 subtype modeled as separate categories with Luminal A as the reference group (Figure 4b, Supporting Information 3), both young age (HR 2.25, 95% CI: 1.27–4.00; *p* = 0.006) and Stage III disease (HR 4.45, 95% CI: 2.41–8.22; *p* < 0.001) remained independent predictors of DFS. However, after incorporating the Sig4 signature (Figure 4c, Supporting Information 4), the continuous Sig4 score remained independently associated with poor DFS (HR 2.45, 95% CI: 1.75–3.44; *p* < 0.001), whereas the estimated association with young age was attenuated and no longer statistically significant (HR 1.50, 95% CI: 0.83–2.70; *p* = 0.176). Stage III disease remained independently associated with worse DFS (HR 3.97, 95% CI: 2.27–6.94; *p* < 0.001). This coefficient attenuation indicates overlapping prognostic information between young age and Sig4 but does not establish mediation.

### 3.6 Descriptive Kaplan–Meier Analysis of DFS According to Sig4 Status in Young Patients

**FIGURE 5:**
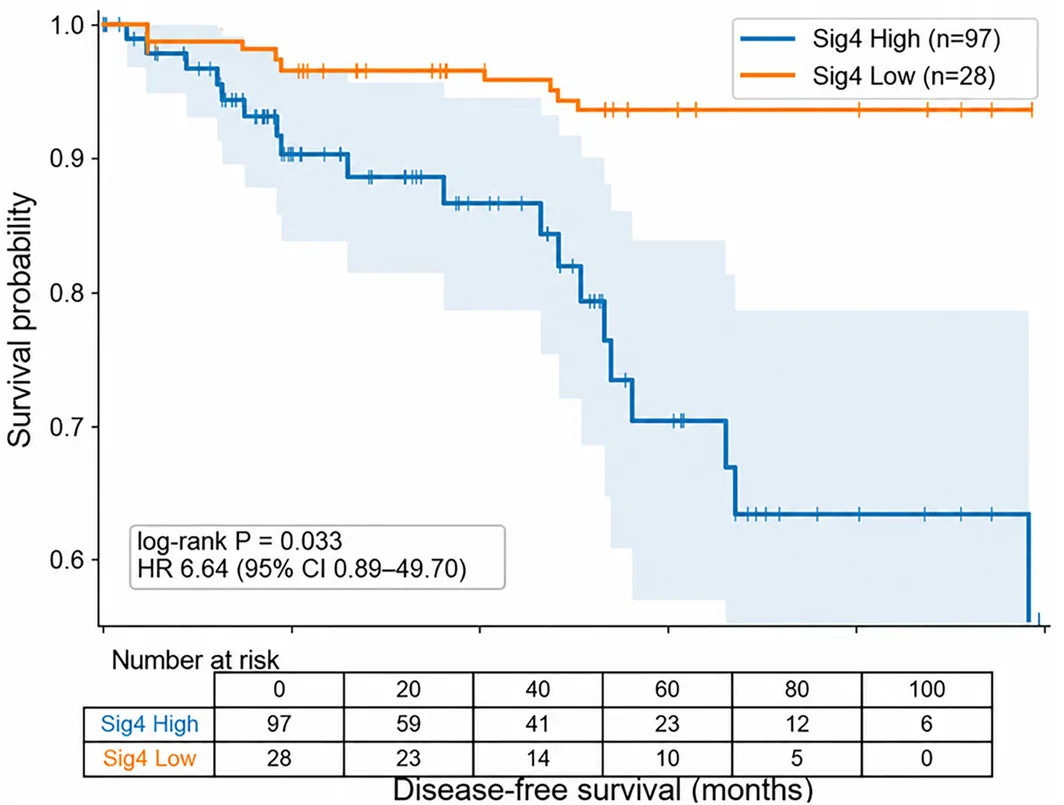
Descriptive Kaplan–Meier analysis of disease‐free survival according to Sig4 status in young patients. Kaplan–Meier curves for disease‐free survival according to Sig4 status (high vs. low) in young patients from The Cancer Genome Atlas cohort. Survival differences were assessed using the log‐rank test. Among young patients with available DFS data, 97 patients were classified as Sig4‐high and 28 as Sig4‐low. DFS events occurred in 19 and 1 patients, respectively. Kaplan–Meier curves include 95% confidence bands, censoring marks, and numbers at risk. Hazard ratios and 95% confidence intervals were estimated with univariable Cox proportional hazards regression, and *p*‐values were obtained with the log‐rank test. This median‐dichotomized analysis was performed for descriptive visualization, whereas the continuous Sig4 score served as the primary analysis in Cox regression.

For visualization, Kaplan–Meier analysis was performed after dichotomizing the Sig4 score at the overall TCGA median. Among the 125 young patients with available DFS data, 97 were classified as Sig4‐high and 28 as Sig4‐low. DFS events occurred in 19 and 1 patients, respectively. Because of the marked imbalance in group size and event counts, this median‐based analysis was considered descriptive, whereas the continuous Sig4 score was treated as the primary analysis. The results revealed that young patients with high Sig4 scores exhibited significantly poorer DFS than those with low Sig4 scores (log‐rank *p* = 0.033; Figure 5).

### 3.7 Multivariable Cox Analysis Within the Young Cohort

**FIGURE 6:**
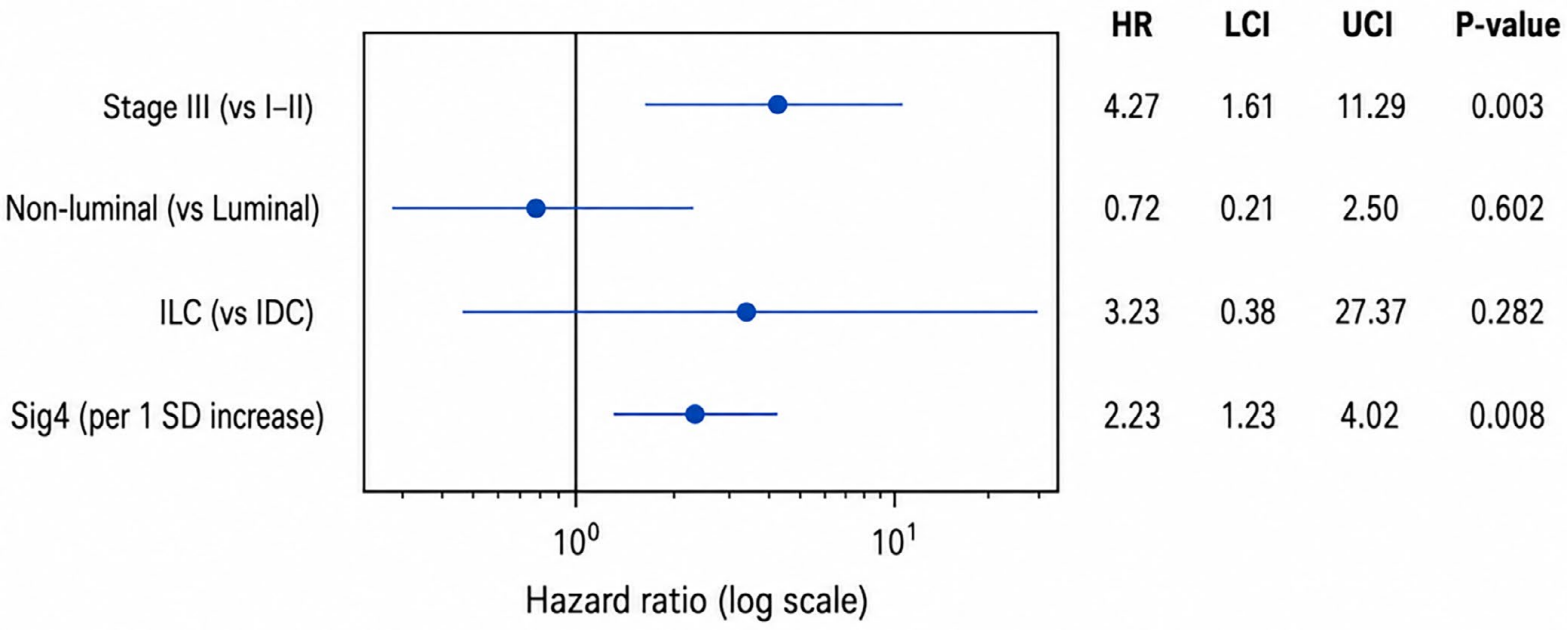
Multivariable Cox proportional hazards analysis for disease‐free survival in the young cohort. Forest plot depicting hazard ratios with 95% confidence intervals from a multivariable Cox proportional hazards model for disease‐free survival in young patients. The model integrated pathological stage, intrinsic subtype, histology, and the Sig4 score modeled as a continuous variable (per 1‐SD increase). Lower and upper confidence intervals are presented as LCI and UCI, respectively. HR, hazard ratio; IDC, invasive ductal carcinoma; ILC, invasive lobular carcinoma; SD, standard deviation.

To further investigate the determinants of DFS in young patients, multivariable Cox proportional hazards analysis was performed (Figure 6, Supporting Information 5). Stage III disease remained significantly associated with worse DFS (HR 3.70, 95% CI: 2.12–6.46; *p* < 0.001). In the conventional multivariable Cox model, the continuous Sig4 score was independently associated with worse DFS (HR 2.23 per 1‐SD increase, model‐based 95% CI: 1.23–4.02; *p* = 0.008). In a nonparametric bootstrap sensitivity analysis with 1000 resamples, the percentile bootstrap 95% CI: for the Sig4 HR was 1.47–4.86. In contrast, neither intrinsic subtype (non‐luminal vs. luminal; HR 0.72, 95% CI: 0.21–2.50; *p* = 0.602) nor histology (ILC vs. IDC; HR 3.23, 95% CI: 0.38–27.37; *p* = 0.282) was significantly associated with DFS in the young subgroup. The model included 20 DFS events and four fitted parameters, corresponding to approximately five events per parameter. The wide CI for histology indicated limited precision, particularly given the small number of ILC cases and events. Accordingly, the findings from this young‐subgroup model should be interpreted as exploratory.

### 3.8 Sequential Cox Proportional Hazards Modeling Incorporating Subtype and Sig4

**FIGURE 7:**
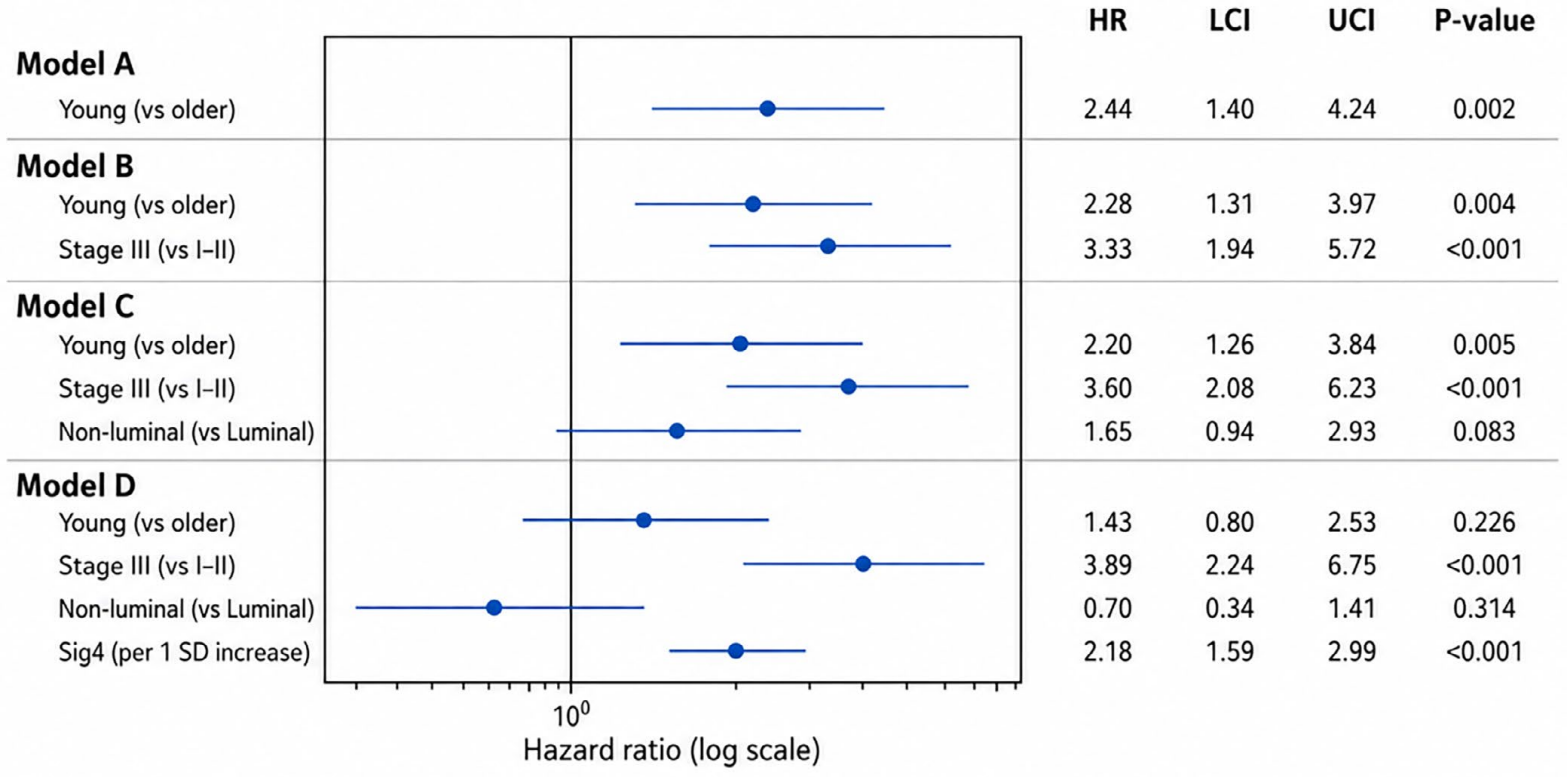
Sequential Cox regression models show that the association between young age and disease‐free survival is attenuated after Sig4 is included. Forest plot depicting hazard ratios with 95% confidence intervals for disease‐free survival across sequential Cox proportional hazards models in The Cancer Genome Atlas cohort. Model A included age group alone; Model B, age group and stage; Model C, age group, stage, and intrinsic subtype; and Model D included age group, stage, intrinsic subtype, and the Sig4 score modeled as a continuous variable per 1‐SD increase. Lower and upper confidence intervals are presented as LCI and UCI, respectively. HR, hazard ratio; SD, standard deviation.

To quantify the prognostic impact of age and the incremental value of the Sig4 score, Cox proportional hazards models were fitted sequentially (Models A–D; Figure 7, Table 3). In the age‐only model (Model A), young age was significantly associated with a diminished DFS (HR 2.44, 95% CI: 1.40–4.24; *p* = 0.002). After adjusting for Stage III disease (Model B), the association with young age was only modestly attenuated and remained significant (HR 2.28, 95% CI: 1.31–3.97; *p* = 0.004). This result indicates that the adverse prognosis observed in young patients cannot be explained solely by disease stage. When intrinsic subtype (non‐luminal vs. luminal) was further incorporated (Model C), the effect of young age remained significant (HR 2.20, 95% CI: 1.26–3.84; *p* = 0.005), whereas the non‐luminal subtype was not independently associated with DFS (HR 1.65, 95% CI: 0.94–2.93; *p* = 0.083). When the Sig4 score was subsequently incorporated as a continuous variable in the fully adjusted model (Model D), a high Sig4 score was independently associated with a poor DFS (HR 2.18 per 1‐SD increase, 95% CI: 1.59–2.99; *p* < 0.001). After including the Sig4 score, the estimated association with young age was attenuated and no longer statistically significant (HR 1.43, 95% CI: 0.80–2.53; *p* = 0.226). This attenuation indicates that the prognostic information from Sig4 partly overlaps with the association between young age and DFS; it should not be interpreted as evidence of mediation or a causal biological explanation.

**TABLE 3:** Proportional hazards models: attenuation of the young‐age effect after incorporating Sig4.

Model	Covariates in the model	Variable	HR (95% CI)	*p*
Model A	Age group	Young (vs. older)	2.44 (1.40–4.24)	0.002
Model B	Age group + stage	Young (vs. older)	2.28 (1.31–3.97)	0.004
Model B		Stage (III vs. I–II)	3.33 (1.94–5.72)	< 0.001
Model C	Age group + stage + subtype	Young (vs. older)	2.20 (1.26–3.84)	0.005
		Stage (III vs. I–II)	3.60 (2.08–6.23)	< 0.001
Model C		Non‐luminal (vs. luminal)	1.65 (0.94–2.93)	0.083
Model D	Age group + stage + subtype + Sig4	Young (vs. older)	1.43 (0.80–2.53)	0.226
		Stage (III vs. I–II)	3.89 (2.2–6.75)	< 0.001
Model D		Nonluminal (vs. luminal)	0.70 (0.34–1.41)	0.314
Model D		Sig4 (per 1 SD increase)	2.18 (1.59–2.99)	< 0.001

Abbreviations: CI, confidence interval; HR, hazard ratio; SD, standard deviation.

### 3.9 Assessment of the Proportional Hazards Assumption in TCGA

The proportional hazards assumption was formally assessed for the reported TCGA Cox regression models with scaled Schoenfeld residual‐based tests. No statistically significant violations were identified in the fully adjusted Model D, the multivariable model restricted to young patients, the PAM50‐adjusted model, or the sensitivity models at age cutoffs of 35, 40, 45, and 50 years. In the intermediate Model C, the nonluminal subtype variable showed possible nonproportionality (*p* = 0.035); however, the global test for this model was not significant (*p* = 0.115). In Model D, neither the individual covariates nor the global test indicated significant nonproportionality (global *p* = 0.276). Full results are provided in Supporting Information 6.

### 3.10 Sensitivity Analyses Using Alternative Age Cutoffs

To address potential arbitrariness in defining “young” breast cancer, the fully adjusted Cox models were reanalyzed using alternative age thresholds: ≤ 35, ≤ 40, ≤ 45, and ≤ 50 years (Table 4). Across all cutoffs, the Sig4 score remained independently associated with a poor DFS, with HRs of 2.28 (95% CI: 1.68–3.10; *p* < 0.001) for ≤ 35 years, 2.22 (95% CI: 1.63–3.03; *p* < 0.001) for ≤ 40 years, 2.19 (95% CI: 1.60–3.01; *p* < 0.001) for ≤ 45 years, and 2.28 (95% CI: 1.66–3.11; *p* < 0.001) for ≤ 50 years. These results indicate that the prognostic association of the fixed Sig4 score remained consistent across alternative age‐group definitions in the final Cox models. However, because the gene‐selection procedure was not repeated for each cutoff, these analyses do not establish that Sig4 was derived independently of the original ≤ 45‐year definition.

**TABLE 4:** Sensitivity analyses using alternative age cutoffs: Independent prognostic impact of Sig4 on DFS.

Age cutoff (years)	Sig4 HR (per 1 SD)	95% CI	*p*
≤ 35 vs. > 35	2.28	1.68–3.10	< 0.001
≤ 40 vs. > 40	2.22	1.63–3.03	< 0.001
≤ 45 vs. > 45	2.19	1.60–3.01	< 0.001
≤ 50 vs. > 50	2.28	1.66–3.11	< 0.001

Abbreviations: CI, confidence interval; DFS, disease‐free survival; HR, hazard ratio; SD, standard deviation.

### 3.11 Hallmark Pathway Enrichment Associated With the Sig4 Signature

**FIGURE 8:**
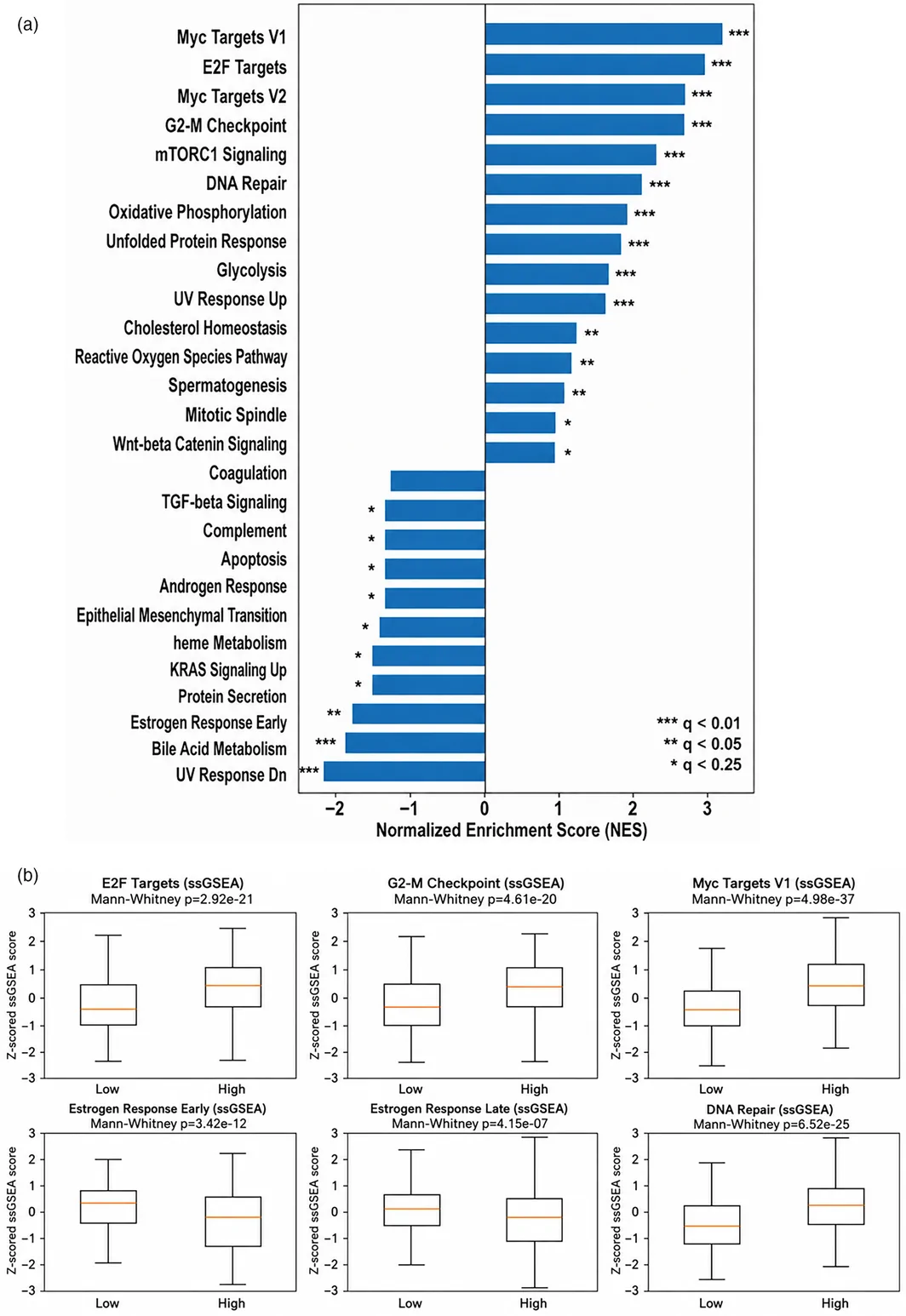
Hallmark pathway enrichment associated with the Sig4 signature. (a) GSEA of Hallmark gene sets comparing Sig4‐high and Sig4‐low tumors in the TCGA young cohort. Pathways with positive normalized enrichment scores were enriched in Sig4‐high tumors, whereas pathways with negative normalized enrichment scores were enriched in Sig4‐low tumors. (b) ssGSEA scores for selected Hallmark pathways in Sig4‐high and Sig4‐low tumors. Sig4‐high tumors showed higher pathway activity for proliferation‐, cell cycle‐, and DNA repair–related programs, whereas Sig4‐low tumors showed higher estrogen response scores. Differences in ssGSEA scores between groups were assessed using the Mann–Whitney *U* test. GSEA, gene set enrichment analysis; ssGSEA, single‐sample GSEA; TCGA, The Cancer Genome Atlas.

To explore the biological programs associated with the Sig4 signature, GSEA was performed using Hallmark gene sets in the TCGA young cohort. Sig4‐high tumors showed enrichment of proliferation‐ and cell cycle–related pathways, including MYC targets, E2F targets, G2–M checkpoint, and mitotic spindle (Figure 8a). In addition, DNA repair, mTORC1 signaling, glycolysis, oxidative phosphorylation, and unfolded protein response were enriched in Sig4‐high tumors. In contrast, Sig4‐low tumors showed relative enrichment of estrogen response pathways, including estrogen response early and estrogen response late. To further evaluate these pathway‐level differences at the individual‐sample level, ssGSEA was performed for selected Hallmark pathways. Consistent with the GSEA results, ssGSEA scores for E2F targets, G2–M checkpoint, MYC targets V1, and DNA repair were significantly higher in Sig4‐high tumors, whereas estrogen response early and estrogen response late scores were higher in Sig4‐low tumors (Figure 8b).

### 3.12 External Validation of Sig4 in the METABRIC Cohort

We then evaluated the external validity of the TCGA‐derived Sig4 signature in the METABRIC cohort. Sig4 scores could be calculated for 1980 METABRIC samples with available expression data. After matching with clinical information, 1134 cases were included in the external validation analysis. The median Sig4 score in the validation cohort was 0.03817, which was used as the cutoff to classify patients into Sig4‐high and Sig4‐low groups, with 567 patients in each group.

In the overall METABRIC validation cohort, young age was associated with a worse RFS in univariable Cox analysis (HR, 1.480; 95% CI: 1.170–1.872; *p* = 0.0011) (Table 5). The TCGA‐derived Sig4 score was also associated with a worse RFS when analyzed as a continuous variable (HR, 1.248; 95% CI: 1.112–1.402; *p* = 0.00018). Consistently, dichotomization by the median Sig4 score showed that the Sig4‐high group had a worse RFS than the Sig4‐low group (HR, 1.337; 95% CI: 1.115–1.603; *p* = 0.0017). In multivariable Cox analysis, both young age and Sig4‐high status remained significantly associated with a worse RFS (Table 6). Young age was associated with an increased risk of relapse (HR, 1.417; 95% CI: 1.112–1.807; *p* = 0.0048), and Sig4‐high status was independently associated with a worse RFS (HR, 1.328; 95% CI: 1.104–1.596; *p* = 0.0025). In the original METABRIC multivariable model, scaled Schoenfeld residual‐based testing indicated non‐proportional hazards for young age (*p* = 0.0016), Stage III disease (*p* = 0.0098), and luminal versus nonluminal subtype (*p* = 1.8 × 10^−9^), resulting in a significant global test (*p* = 8.0 × 10^−9^). In contrast, no such evidence was observed for Sig4‐high status (*p* = 0.704) or histology (*p* = 0.459).

**TABLE 5:** Univariable Cox regression analysis of Sig4 status and young age for relapse‐free survival in the METABRIC validation cohort.

Variable	HR	LCI	UCI	*p*
Sig4 score (continuous)	1.248	1.112	1.402	< 0.001
Sig4 high vs. low	1.337	1.115	1.603	< 0.001
Young vs. older	1.480	1.170	1.872	< 0.001

Abbreviations: HR: hazard ratio; LCI, lower confidence interval; UCI, upper confidence interval.

**TABLE 6:** Multivariable Cox regression analysis including young age and Sig4 status for relapse‐free survival in the METABRIC validation cohort.

Variable	HR	LCI	UCI	*p*
Young	1.417	1.112	1.807	0.005
Sig4 high	1.328	1.104	1.600	0.003
Stage III	2.712	2.085	3.527	< 0.001
Nonluminal	1.096	0.906	1.324	0.346
ILC	0.982	0.689	1.402	0.922

Abbreviations: HR, hazard ratio; ILC, invasive lobular carcinoma; LCI, lower confidence interval; UCI, upper confidence interval.

Therefore, a stratified Cox sensitivity analysis was performed, allowing separate baseline hazards according to stage and subtype. In this stratified model, Sig4‐high status remained significantly associated with worse RFS (HR 1.334, 95% CI: 1.110–1.603; *p* = 0.002), and no evidence of non‐proportionality was observed for this variable (*p* = 0.651). The global test was not significant (*p* = 0.128), although residual non‐proportionality was observed for young age (*p* = 0.039). A further model incorporating a time‐varying coefficient for young age suggested attenuation of the age‐associated effect over follow‐up, although the time‐dependent term did not reach conventional statistical significance (*p* = 0.065). Sig4‐high status remained significantly associated with worse RFS in this model (HR 1.337, 95% CI: 1.113–1.607; *p* = 0.002). Full results of the time‐varying coefficient sensitivity analysis are provided in Supporting Information 7.

We further examined whether Sig4 could stratify prognosis specifically in young METABRIC patients aged ≤ 45 years. This subgroup included 162 patients, of whom 85 experienced RFS events. In this young subgroup, however, the Sig4 score was not significantly associated with RFS when analyzed as a continuous variable per 1‐SD increase (HR, 1.009; 95% CI: 0.783–1.300; *p* = 0.945). Thus, external validation in METABRIC supported the prognostic relevance of the TCGA‐derived Sig4 signature in the overall validation cohort, whereas additional prognostic stratification in young patients alone was not confirmed.

## 4 Discussion

Using TCGA data, the present study identified clinicopathological differences between the young and older breast cancer age groups, with significantly lower ILC frequency and more frequent lymph node metastasis observed in young patients than in older ones. The decreased proportion of ILC is consistent with prior reports demonstrating that ILC is more common in older patients than in younger ones [17, 18]. Moreover, the elevated rate of node‐positive disease in young patients also aligns with the results of previous studies [8, 10, 11]. Although OS and DSS did not differ significantly in the present study, young‐onset breast cancer, defined as age ≤ 45 years, was associated with an inferior DFS. This disadvantage was evident in both relative and absolute terms: young patients exhibited lower 5‐ and 10‐year DFSs, higher corresponding event rates, and a significantly shorter RMST than older patients. The larger RMST difference at 10 years than at 5 years further indicates that the prognostic gap widened over time, suggesting an increasing absolute burden of DFS events in young patients.

A key question was whether this adverse prognosis reflected an independent age effect or can be largely explained by differences in stage and molecular subtype. In the present analyses, the adverse DFS associated with young age persisted after simultaneously adjusting for stage and intrinsic subtype. These findings indicate that such conventional determinants do not fully account for the risk of recurrence in young patients, consistent with previous reports [3, 19]. Moreover, Stage III disease remained a strong and independent predictor of DFS across all models, reflecting the established prognostic importance of stage in breast cancer. Although adjusting for stage alone did not eliminate the adverse association between young age and DFS [10, 19], incorporation of the Sig4 score attenuated this association and rendered it statistically non‐significant. In addition, descriptive Kaplan–Meier analysis showed poorer DFS in young patients with high Sig4 scores, while multivariable Cox analysis with the continuous Sig4 score indicated that Sig4 remained independently associated with DFS after adjustment for pathological stage, intrinsic subtype, and histology. Collectively, these findings indicate that Sig4 provides prognostic information that partly overlaps with the association between young age and DFS. However, attenuation of the young‐age coefficient after inclusion of Sig4 does not establish mediation or demonstrate that Sig4 explains the adverse prognosis associated with young age. Such attenuation may reflect correlation between age and Sig4, reduced statistical precision, overadjustment, residual or unmeasured confounding, or other forms of model dependence. The findings should therefore be interpreted as evidence of an association between Sig4 and recurrence‐related outcomes rather than as evidence of a causal or mechanistic relationship. To address the potential arbitrariness of the age definition applied in this study, the fully adjusted Cox models were reanalyzed using alternative age cutoffs of ≤ 35, ≤ 40, ≤ 45, and ≤ 50 years. Across the alternative age cutoffs, the fixed Sig4 score remained independently associated with DFS (*p* < 0.001 for all; Table 4). These sensitivity analyses show that the prognostic association of the fixed Sig4 score did not depend on defining the age covariate exclusively at 45 years. However, because the gene‐selection procedure was not repeated at each alternative cutoff, these analyses do not establish that the derivation of Sig4 itself was independent of the original age definition. Given the lack of a universally accepted age cutoff for defining young‐onset breast cancer, variable cutoffs of 35, 40, or 45 years have been used across studies. Therefore, in this study, an age of 45 years was selected as the pragmatic cutoff, while the other thresholds were examined in sensitivity analyses. The findings remained consistent across these age thresholds in the final Cox models; however, this consistency applies only to the prognostic association of the fixed Sig4 score and not to the stability of the gene‐selection process itself.

Although the distinctive molecular characteristics of young‐onset breast cancer have been previously reported [2, 3, 15, 20, 21, 22, 23], the present study explored whether age‐associated transcriptional differences could be summarized as a compact signature associated with recurrence‐related outcomes. By intersecting age‐associated expression changes with DFS‐associated signals, the analysis identified a four‐gene signature with potential prognostic relevance. However, these findings do not establish that Sig4 captures the biological basis of young‐onset breast cancer or that it represents a clinically applicable biomarker. Rather, Sig4 should be regarded as an exploratory expression signature whose prognostic association requires further validation in independent prospective cohorts. This outcome‐focused selection strategy differs from previous studies that primarily characterized the broader transcriptomic features of young‐onset breast cancer, because it identified molecular features associated with both age group and DFS rather than the full biological spectrum of tumors arising in young patients. Recent breast cancer prognostic studies have developed models from biologically predefined gene sets, including relaxin‐related, Kbhb‐associated, and mitochondria‐related genes, and have compared multiple machine‐learning algorithms across training and validation cohorts [24, 25, 26]. Other studies have combined prognostic analysis with single‐cell profiling, spatial transcriptomics, gene perturbation, or in vivo experiments to connect candidate genes with specific functional states [25, 27]. Clinical prediction research has also emphasized training–validation separation, calibration, decision‐curve analysis, and interpretable approaches such as SHAP [28]. In contrast, Sig4 was not developed as an optimized clinical prediction model. It differs methodologically by restricting candidate genes to those associated with both age group and recurrence‐related outcome before penalized and multivariable Cox selection, resulting in a compact four‐gene signature derived from a clinically defined age‐related contrast. Applying the fixed TCGA‐derived genes and coefficients to METABRIC provided partial evidence of transportability, but the lack of confirmation in the young METABRIC subgroup indicates that generalizability specifically to young patients remains unproven. Notably, the heterogeneous functional composition of Sig4 is not necessarily inconsistent with prior literature. Instead, it may reflect the multifactorial nature of a poor prognosis in young patients, which cannot be reduced to a single pathway, such as proliferation or stemness alone. Consistent with this interpretation, the four component genes included in Sig4 were not driven by a single dominant transcript but instead exhibited coordinated differences across Sig4‐high and Sig4‐low tumors in young patients. Overall, these findings suggest that Sig4 is associated with a composite transcriptional pattern observed in tumors with adverse outcomes. The Sig4 model assigns a positive coefficient to *NOA1* and negative coefficients to *LINC01124*, *ZNF704*, and *AGFG2*; therefore, Sig4‐high status reflects a composite expression configuration rather than a uniform upregulation of all four genes. C4orf14 (*NOA1*) is a mitochondrial GTPase involved in mitoribosome biogenesis, mitochondrial protein synthesis, mitochondrial respiration, and apoptosis [29, 30, 31]. *LINC01124* is a long noncoding RNA implicated in proliferation, migration, and invasion through miRNA‐mediated pathways in several cancer types; however, breast cancer‐specific evidence remains limited [32, 33, 34]. *ZNF704* is a zinc finger transcriptional repressor linked in breast cancer to SIN3A‐associated repression of PER2, circadian disruption, and enhanced proliferation and metastasis [35, 36]. *AGFG2* participates in stimulus‐dependent secretion and vesicular trafficking, potentially reflecting tumor–microenvironment interactions [37]. Collectively, the known functions of these four genes involve distinct biological processes, including mitochondrial regulation, post‐transcriptional regulation, transcriptional repression, and vesicular trafficking. These observations provide a possible biological context for the Sig4‐associated transcriptional pattern but do not establish that Sig4 represents an integrated tumor functional state. The pathway‐level analyses further support this interpretation. GSEA showed that Sig4‐high tumors were enriched in MYC targets, E2F targets, G2–M checkpoint, mitotic spindle, DNA repair, mTORC1 signaling, glycolysis, oxidative phosphorylation, and unfolded protein response. This indicates that Sig4‐high status was associated not only with proliferation‐related pathways but also with transcriptional programs involving cell‐cycle activity, metabolism, DNA repair, and cellular stress responses. The ssGSEA analysis further confirmed that several of these pathway differences were also evident at the individual‐sample level. In contrast, estrogen response pathways were relatively enriched in Sig4‐low tumors, suggesting that the biological contrast captured by Sig4 may also involve attenuation of estrogen‐responsive transcriptional programs in Sig4‐high tumors. The pathway profile associated with Sig4 also partly overlaps with recent functionally oriented breast cancer studies linking adverse prognosis to mitochondrial and lipid metabolic reprogramming, Kbhb‐associated metabolic states, R‐loop regulation, cancer stemness, and altered immune–metabolic programs [24, 25, 26, 27]. Consistent with these reports, Sig4‐high tumors were enriched for cell‐cycle, DNA‐repair, glycolytic, oxidative‐phosphorylation, mTORC1, and stress–response pathways. However, this convergence should be interpreted at the pathway level only. Unlike studies incorporating single‐cell localization, metabolic assays, gene perturbation, or in vivo validation [25, 26, 27], the present analysis was based on bulk transcriptomic associations. Therefore, the enrichment results support the biological plausibility of Sig4 as a marker of an adverse transcriptional phenotype but do not establish that the four component genes directly regulate these pathways or constitute a unified mechanism of young‐onset breast cancer. Taken together, these pathway analyses offer a descriptive biological context for the observed prognostic association of Sig4. However, because the analyses were correlative, they do not establish that Sig4 represents a specific tumor functional state or mechanistically contributes to recurrence. The interpretation of Sig4 should also be placed within the rapidly evolving breast cancer biomarker landscape. Contemporary biomarker development is moving beyond static assessment of ER, PR, and HER2 toward dynamic and multimodal characterization of tumor biology. Clinically actionable genomic alterations, including those in PIK3CA, ESR1, and BRCA1/2, can inform treatment selection in defined therapeutic contexts, whereas immune‐ and tumor microenvironment‐associated biomarkers may provide complementary information regarding treatment response. Liquid biopsy approaches, particularly circulating tumor DNA analysis, further enable longitudinal monitoring of molecular evolution, emerging resistance, and minimal residual disease. In parallel, the integration of genomic, transcriptomic, proteomic, spatial, imaging, and clinical information is increasingly viewed as necessary to capture the heterogeneity and temporal evolution of breast cancer [38].

In this context, Sig4 represents a static, tissue‐based transcriptomic signature derived from bulk tumor samples rather than a dynamic, multimodal, or directly actionable biomarker. Its pathway associations may reflect both tumor‐intrinsic transcriptional programs and contributions from the tumor microenvironment, but the present data do not distinguish these components. Translation of Sig4 into a clinically applicable biomarker would therefore require additional steps, including analytical standardization across assay platforms, a predefined and transferable scoring method and cutoff, calibration in independent cohorts, prospective validation, and demonstration that its use improves clinical decision‐making beyond established clinicopathological and molecular biomarkers. Future studies could also examine whether Sig4 provides complementary information when integrated with actionable genomic alterations, immune or spatial biomarkers, and longitudinal liquid biopsy measurements. Until such evidence is available, Sig4 should be regarded as an exploratory prognostic association rather than a clinically implemented or treatment‐directing biomarker. External validation in METABRIC provided partial support for the prognostic relevance of the TCGA‐derived Sig4 signature. The four‐gene composition and the TCGA‐derived regression coefficients were fixed before application to METABRIC and were not re‐estimated in the validation cohort. Despite differences in expression platform and data preprocessing, Sig4 remained associated with worse RFS in the overall METABRIC cohort when evaluated both as a continuous score and as a variable dichotomized at the median. The association of Sig4‐high status was also maintained after stage and subtype were handled with stratified Cox regression and after the time‐dependent effect of young age was considered. These sensitivity analyses support the stability of this prognostic association in the overall METABRIC cohort under alternative model specifications. However, external validation was not uniformly successful. When the analysis was restricted to METABRIC patients aged ≤ 45 years, the Sig4 score was not associated with RFS (HR per 1‐SD increase, 1.009; 95% CI: 0.783–1.300; *p* = 0.945). However, the absence of a significant association within the METABRIC subgroup aged ≤ 45 years indicates that its prognostic utility specifically for young‐onset breast cancer was not externally validated. The absence of an association in the young subgroup may reflect limited precision, differences in cohort composition, treatment background, expression platform, preprocessing, and endpoint definition, or a lack of reproducibility of the young‐specific prognostic association. TCGA used DFS, whereas METABRIC used RFS. Although both endpoints capture recurrence‐related outcomes, their database‐specific definitions and included events are not necessarily equivalent. The METABRIC analysis should therefore be interpreted as validation of the association of Sig4 with a related recurrence endpoint rather than as an exact replication of the TCGA DFS analysis. In addition, TCGA used RNA‐seq data, whereas METABRIC used microarray‐based expression measurements, and the gene expression values required cohort‐level standardization before the Sig4 score was calculated. These differences may have affected the transportability and calibration of the score across datasets. The external validation should thus be regarded as partial rather than definitive. Although the overall METABRIC results reduce the likelihood that Sig4 represents a purely TCGA‐specific finding, they do not establish its clinical utility in young‐onset breast cancer. Validation in independent cohorts enriched for young patients, with harmonized recurrence endpoints, standardized expression assays, predefined score calculation procedures, and detailed treatment annotation, is required before Sig4 can be considered for risk stratification specific to young patients. The failure to reproduce prognostic stratification within the young METABRIC subgroup substantially limits the proposed clinical utility of Sig4 in young patients.

The observed associations should be interpreted in the context of incomplete treatment and hereditary predisposition information in TCGA. Younger patients and those with more aggressive tumors may have been more likely to receive intensive systemic therapy. Chemotherapy could have reduced recurrence risk in younger patients with high‐risk disease, thereby attenuating the observed adverse association of young age or Sig4 with DFS. Similarly, endocrine therapy may have modified recurrence risk in hormone receptor‐positive tumors, whereas HER2‐targeted therapy may have substantially altered outcomes in HER2‐positive disease. Conversely, incomplete, heterogeneous, or delayed receipt of indicated therapy could have exaggerated recurrence differences between patient groups. Germline cancer predisposition, including BRCA1/2‐associated disease, may also have been more prevalent among younger patients and could have been associated with both tumor phenotype and recurrence risk. In addition, changes in systemic treatment standards over the treatment era represented in TCGA may have influenced outcomes, particularly, for hormone receptor‐positive and HER2‐positive tumors. Because germline status, treatment type, treatment intensity, adherence, and treatment era were not consistently documented, we could not determine whether the observed associations reflected tumor biology alone or were partly influenced by these factors. The longer follow‐up in young patients may have increased the opportunity to detect DFS events. Although time‐to‐event methods account for censoring, these factors may still have influenced the magnitude of the observed associations. This study has several limitations. First, it was a retrospective analysis of publicly available datasets and was therefore subject to the inherent limitations of retrospective observational research, including selection bias, incomplete clinical annotation, unmeasured confounding, and heterogeneity in treatment and follow‐up. Detailed information on endocrine therapy, chemotherapy, and HER2‐targeted therapy was not consistently available in TCGA. DFS information was unavailable for 10.5% of the cohort, and median follow‐up differed between age groups. These factors may have affected the magnitude, precision, and interpretation of the observed survival associations.

Second, the limited number of DFS events relative to the number of genes evaluated increased the possibility of model overfitting and unstable feature selection, particularly in the young‐subgroup analyses. Although LASSO‐Cox regression with cross‐validation was used for penalized variable reduction and the model was subsequently reduced to a parsimonious four‐gene signature, these procedures reduce but do not eliminate the risk of overfitting. The additional AIC‐based stepwise selection and estimation of the final coefficients within the same TCGA derivation dataset may have resulted in optimistic estimates of model performance. Therefore, Sig4 should be regarded as an exploratory candidate signature rather than a fully validated prognostic model. Third, the analyses were based on bulk RNA‐seq data; therefore, the observed Sig4‐associated transcriptional patterns may reflect both tumor cell–intrinsic programs and contributions from the tumor microenvironment. Fourth, although GSEA and ssGSEA provided a biological context for the Sig4 signature, these analyses are inherently correlative and cannot establish causal relationships between Sig4 and the enriched pathways. In addition, the GSEA analysis based on Sig4‐high and Sig4‐low groups may be influenced by the median cutoff used for dichotomization. Future studies using independent cohorts, continuous score‐based pathway analyses, and experimental validation are needed to clarify the biological mechanisms underlying the Sig4‐associated high‐risk phenotype. Fifth, although the prognostic relevance of the TCGA‐derived Sig4 signature was externally evaluated in the METABRIC cohort, differences in expression profiling platforms, preprocessing procedures, endpoint definitions, and available clinical annotations limit direct comparison between TCGA and METABRIC. TCGA used DFS, whereas METABRIC validation relied on RFS. Although both are recurrence‐related endpoints, their database‐specific definitions and event components may differ, limiting direct comparability between the two cohorts. Therefore, the METABRIC analysis represents validation with a related, but not identical, clinical endpoint. TCGA used RNA‐seq data, whereas METABRIC used microarray‐based expression measurements and required cohort‐specific preprocessing and standardization. Moreover, the ability of Sig4 to further stratify prognosis in young METABRIC patients was not confirmed, possibly owing to differences in cohort composition, endpoint definition, and sample size. Moreover, the proportional hazards assumption was not uniformly satisfied in the METABRIC analyses, particularly for young age, stage, and luminal versus nonluminal subtype. This indicates that the magnitude of these associations varied over time and that their constant HRs should be interpreted as average effects over the follow‐up period. Stratified and time‐varying coefficient sensitivity analyses were therefore performed. Although the association between Sig4‐high status and worse RFS remained significant in these analyses, residual model dependence is an important limitation. Therefore, the Sig4 signature should still be regarded as an exploratory candidate requiring further validation in independent young breast cancer cohorts with harmonized expression platforms and sufficient recurrence‐related outcome data.

The present study identified Sig4 as an exploratory age‐associated four‐gene expression signature linked to recurrence‐related outcomes in breast cancer. The association was observed with DFS in TCGA and with RFS in the overall METABRIC cohort, but was not confirmed within the young METABRIC subgroup. Accordingly, the present findings support the potential general prognostic association of Sig4 in breast cancer but do not establish its utility for stratification specifically in young‐onset disease. Independent validation in prospective cohorts enriched for young patients is required.

## Author Contributions

**Shiro Uchida:** conceptualization, methodology, data curation, investigation, validation, formal analysis, supervision, resources, project administration, visualization, writing – original draft, writing – review and editing.

## Funding

The author has nothing to report.

## Ethics Statement

The author has nothing to report.

## Consent

The author has nothing to report.

## Conflicts of Interest

The author declares no conflicts of interest.

## Supporting information


**Supporting Information: 1.** Cox regression coefficients used to calculate the Sig4 score.

**Supporting Information: 2.** Univariable Cox proportional hazards regression analysis for disease‐free survival.

**Supporting Information: 3.** Multivariable Cox proportional hazards regression analysis of clinicopathological variables for disease‐free survival.

**Supporting Information: 4.** Multivariable Cox proportional hazards regression analysis including the Sig4 score for disease‐free survival.

**Supporting Information: 5.** Multivariable Cox proportional hazards analysis for disease‐free survival in the young cohort.

**Supporting Information: 6.** Proportional hazards assumption testing for the reported Cox regression models.

**Supporting Information: 7.** Time‐varying coefficient sensitivity analysis in the METABRIC cohort.

## Data Availability

All datasets analyzed in this study are publicly available from TCGA and cBioPortal. Processed data and analysis scripts are available from the corresponding author upon reasonable request. No new datasets were generated during the current study.

## References

[1] F. Bray , M. Laversanne , H. Sung , et al., “Global Cancer Statistics 2022: GLOBOCAN Estimates of Incidence and Mortality Worldwide for 36 Cancers in 185 Countries,” CA: A Cancer Journal for Clinicians 74 (2024): 229–263, 10.3322/caac.21834. 38572751

[2] C. K. Anders , D. S. Hsu , G. Broadwater , et al., “Young Age at Diagnosis Correlates With Worse Prognosis and Defines a Subset of Breast Cancers With Shared Patterns of Gene Expression,” Journal of Clinical Oncology 26 (2008): 3324–3330, 10.1200/JCO.2007.14.2471. 18612148

[3] H. A. Azim, Jr. , S. Michiels , P. L. Bedard , et al., “Elucidating Prognosis and Biology of Breast Cancer Arising in Young Women Using Gene Expression Profiling,” Clinical Cancer Research 18 (2012): 1341–1351, 10.1158/1078-0432.CCR-11-2599. 22261811

[4] J. Fu , L. Wu , T. Xu , et al., “Young‐Onset Breast Cancer: A Poor Prognosis Only Exists in Low‐Risk Patients,” Journal of Cancer 10 (2019): 3124–3132, 10.7150/jca.30432. 31289582 PMC6603374

[5] H. O. Adami , B. Malker , L. Holmberg , I. Persson , and B. Stone , “The Relation Between Survival and Age at Diagnosis in Breast Cancer,” New England Journal of Medicine 315 (1986): 559–563, 10.1056/NEJM198608283150906. 3736639

[6] J. Kollias , C. W. Elston , I. O. Ellis , J. F. Robertson , and R. W. Blamey , “Early‐Onset Breast Cancer—Histopathological and Prognostic Considerations,” British Journal of Cancer 75 (1997): 1318–1323, 10.1038/bjc.1997.223. 9155052 PMC2228226

[7] A. E. Arikan , H. Kara , O. Dülgeroğlu , E. N. Erdoğan , E. Capkinoglu , and C. Uras , “Do Prognosis and Clinicopathological Features Differ in Young Early‐Stage Breast Cancer?,” Frontiers in Surgery 9 (2022): 900363, 10.3389/fsurg.2022.900363. 36338611 PMC9629693

[8] A. Bharat , R. L. Aft , F. Gao , and J. A. Margenthaler , “Patient and Tumor Characteristics Associated With Increased Mortality in Young Women (≤40 Years) With Breast Cancer (<or =40 Years) With Breast Cancer,” Journal of Surgical Oncology 100 (2009): 248–251, 10.1002/jso.21268. 19330813

[9] N. S. El Saghir , M. Seoud , M. K. Khalil , et al., “Effects of Young Age at Presentation on Survival in Breast Cancer,” BMC Cancer 6 (2006): 194, 10.1186/1471-2407-6-194. 16857060 PMC1555600

[10] J. L. Gnerlich , A. D. Deshpande , D. B. Jeffe , A. Sweet , N. White , and J. A. Margenthaler , “Elevated Breast Cancer Mortality in Women Younger Than Age 40 Years Compared With Older Women Is Attributed to Poorer Survival in Early‐Stage Disease,” Journal of the American College of Surgeons 208 (2009): 341–347, 10.1016/j.jamcollsurg.2008.12.001. 19317994 PMC3262236

[11] A. J. Nixon , D. Neuberg , D. F. Hayes , et al., “Relationship of Patient Age to Pathologic Features of the Tumor and Prognosis for Patients With Stage I or II Breast Cancer,” Journal of Clinical Oncology 12 (1994): 888–894, 10.1200/JCO.1994.12.5.888. 8164038

[12] Z. Liu , Z. Sahli , Y. Wang , A. C. Wolff , L. M. Cope , and C. B. Umbricht , “Young Age at Diagnosis Is Associated With Worse Prognosis in the Luminal A Breast Cancer Subtype: A Retrospective Institutional Cohort Study,” Breast Cancer Research and Treatment 172 (2018): 689–702, 10.1007/s10549-018-4950-4. 30225619 PMC6786966

[13] K. A. Hoadley , C. Yau , T. Hinoue , et al., “Cell‐of‐Origin Patterns Dominate the Molecular Classification of 10,000 Tumors From 33 Types of Cancer,” Cell 173 (2018): 291–304.e6, 10.1016/j.cell.2018.03.022. 29625048 PMC5957518

[14] R. R. Bastien , Á. Rodríguez‐Lescure , M. T. Ebbert , et al., “PAM50 Breast Cancer Subtyping by RT‐qPCR and Concordance With Standard Clinical Molecular Markers,” BMC Medical Genomics 5 (2012): 44, 10.1186/1755-8794-5-44. 23035882 PMC3487945

[15] H. A. Azim, Jr. , B. Nguyen , S. Brohée , G. Zoppoli , and C. Sotiriou , “Genomic Aberrations in Young and Elderly Breast Cancer Patients,” BMC Medicine 13 (2015): 266, 10.1186/s12916-015-0504-3. 26467651 PMC4606505

[16] P. Selenica , H. Dopeso , M. Repetto , et al., “Genomic Landscape of Breast Cancer in Elderly Patients,” npj Breast Cancer 11 (2025): 70, 10.1038/s41523-025-00781-4. 40640183 PMC12246064

[17] H. Abdel‐Razeq , S. Iweir , R. Abdel‐Razeq , et al., “Differences in Clinicopathological Characteristics, Treatment, and Survival Outcomes Between Older and Younger Breast Cancer Patients,” Scientific Reports 11 (2021): 14340, 10.1038/s41598-021-93676-w. 34253800 PMC8275803

[18] S. Uchida , “Age‐Stratified Analysis of Invasive Lobular Carcinoma Reveals Comparable Clinicopathological and Molecular Features Across Age Groups,” Discover Oncology 17 (2026): 477, 10.1007/s12672-026-04700-2. 41706335 PMC13022215

[19] A. H. Partridge , M. E. Hughes , E. T. Warner , et al., “Subtype‐Dependent Relationship Between Young Age at Diagnosis and Breast Cancer Survival,” Journal of Clinical Oncology 34 (2016): 3308–3314, 10.1200/JCO.2015.65.8013. 27480155

[20] R. O. C. Humlevik , A. A. Svanøe , T. Aas , et al., “Distinct Clinicopathological Features and Treatment Differences in Breast Cancer Patients of Young Age,” Scientific Reports 15 (2025): 5655, 10.1038/s41598-025-90053-9. 39955428 PMC11830014

[21] H. A. Azim, Jr. and A. H. Partridge , “Biology of Breast Cancer in Young Women,” Breast Cancer Research 16 (2014): 427, 10.1186/s13058-014-0427-5. 25436920 PMC4303229

[22] R. H. Johnson , P. Hu , C. Fan , and C. K. Anders , “Gene Expression in “Young Adult Type” Breast Cancer: A Retrospective Analysis,” Oncotarget 6 (2015): 13688–13702, 10.18632/oncotarget.4051. 25999348 PMC4537042

[23] L. M. Ingebriktsen , K. Finne , L. A. Akslen , and E. Wik , “A Novel Age‐Related Gene Expression Signature Associates With Proliferation and Disease Progression in Breast Cancer,” British Journal of Cancer 127 (2022): 1865–1875, 10.1038/s41416-022-01953-w. 35995935 PMC9643541

[24] Y. Du , Q. Yuan , H. Yu , et al., “Utilization of Machine Learning Algorithms for the Identification of the RLN Associated Prognostic Model and Feature Biomarkers of RLN‐Related Subtypes in Breast Cancer,” Translational Oncology 65 (2026): 102684, 10.1016/j.tranon.2026.102684. 41581316 PMC12860642

[25] Q. Yuan , Y. Sha , R. Ye , et al., “Machine Learning‐Based Identification of Kbhb‐Affected Tumor Cell Subsets as Prognostic and Therapeutic Targets in Breast Cancer,” Journal of Translational Medicine 24 (2026): 71, 10.1186/s12967-025-07555-3. PMC12802299 41382084

[26] Y. Sha , Y. Du , M. Niu , Q. Yuan , and J. Han , “Mitochondrial Gene APOO Reprograms Lipid Metabolism to Influence the Prognosis of Breast Cancer,” Translational Cancer Research 15 (2026): 294, 10.21037/tcr-2025-aw-2554. 42180875 PMC13191036

[27] Q. Yuan , H. Zhang , N. Zhang , et al., “BPHL Promotes TNBC Stemness by Resolving R‐Loops via POLR2A Lactylation Inhibition and BARD1‐Mediated Ubiquitination,” Cancer Letters 645 (2026): 218388, 10.1016/j.canlet.2026.218388. 41765360

[28] Q. Yuan , R. Ye , Y. Qian , et al., “Machine Learning and SHAP Value Interpretation for Predicting the Response to Neoadjuvant Chemotherapy and Long‐Term Clinical Outcomes in Chinese Female Breast Cancer,” Annals of Medicine 57 (2025): 2541316, 10.1080/07853890.2025.2541316. 40754951 PMC12322993

[29] J. He , H. M. Cooper , A. Reyes , et al., “Human C4orf14 Interacts With the Mitochondrial Nucleoid and Is Involved in the Biogenesis of the Small Mitochondrial Ribosomal Subunit,” Nucleic Acids Research 40 (2012): 6097–6108, 10.1093/nar/gks257. 22447445 PMC3401442

[30] M. Kolanczyk , M. Pech , T. Zemojtel , et al., “NOA1 Is an Essential GTPase Required for Mitochondrial Protein Synthesis,” Molecular Biology of the Cell 22 (2011): 1–11, 10.1091/mbc.E10-07-0643. 21118999 PMC3016967

[31] T. Tang , B. Zheng , S. H. Chen , et al., “hNOA1 Interacts With Complex I and DAP3 and Regulates Mitochondrial Respiration and Apoptosis,” Journal of Biological Chemistry 284 (2009): 5414–5424, 10.1074/jbc.M807797200. 19103604 PMC2643507

[32] Z. B. Wang , H. Y. Zhang , and J. B. Lu , “Expression and Effects of Long Non‐Coding RNA, LINC01124, in Non‐Small Cell Lung Cancer,” Oncotargets and Therapy 12 (2019): 11729–11736, 10.2147/OTT.S214049. 32099381 PMC6997214

[33] Y. J. Wu , Z. Q. Cai , R. M. He , X. C. Wang , L. L. Cong , and F. H. Qiu , “Knockdown of Long Noncoding RNA 01124 Inhibits the Malignant Behaviors of Colon Cancer Cells via miR‐654‐5p/HAX‐1,” Evidence‐Based Complementary and Alternative Medicine 2022 (2022): 1–10, 10.1155/2022/1092107.

[34] L. Sun , Y. Zhang , Y. Yao , H. Du , Y. Zhang , and A. Fang , “Long Noncoding RNA LINC01124 Activates Hepatocellular Carcinoma Cell Proliferation, Migration, and Invasion by Absorbing microRNA‐1247‐5p and Overexpressing FOXO3,” Oncology Research 29 (2021): 175–187, 10.32604/or.2022.03550. 37304672 PMC10208059

[35] C. Yang , J. Wu , X. Liu , et al., “Circadian Rhythm Is Disrupted by ZNF704 in Breast Carcinogenesis,” Cancer Research 80 (2020): 4114–4128, 10.1158/0008-5472.CAN-20-0493. 32651256

[36] D. Wang , S. Yang , M. Lyu , L. Xu , S. Zhong , and D. Yu , “Circular RNA HSDL2 Promotes Breast Cancer Progression via miR‐7978 ZNF704 Axis and Regulating Hippo Signaling Pathway,” Breast Cancer Research 26 (2024): 105, 10.1186/s13058-024-01864-z. 38937788 PMC11210124

[37] A. Watanabe , H. Hataida , N. Inoue , et al., “Arf GTPase‐Activating Proteins SMAP1 and AGFG2 Regulate the Size of Weibel‐Palade Bodies and Exocytosis of von Willebrand Factor,” Biology Open 10 (2021): bio058789, 10.1242/bio.058789. 34369554 PMC8430232

[38] D. F. Dai and T. Micsik , “Novel Biomarkers in Breast Cancer for Precision Therapy and Adaptive Treatment Strategies,” Advances in Translational Research 1 (2026): 179–192, 10.1556/1661.2026.00016.

