# Supplementary material for: Age‐Associated Four‐Gene Prognostic Signature in Breast Cancer
Source: Cancer Rep (Hoboken). 2026 Sep 1;9(9):e70670. doi: 10.1002/cnr2.70670 (PMC13533818; doi:10.1002/cnr2.70670)
Supplement: Supplementary file 1 [file CNR2-9-e70670-s001.docx]

**Supporting information 2: Univariable Cox proportional hazards regression analysis for disease-free survival.**

| Variable | HR (95% CI) | P value |
| --- | --- | --- |
| Age group: Young vs Older | 2.44 (1.40-4.24) | 0.002 |
| Histology: ILC vs IDC | 0.83 (0.39-1.75) | 0.618 |
| Luminal B vs Luminal A | 1.07 (0.51-2.22) | 0.867 |
| Her2 vs Luminal | 1.61 (0.66-3.93) | 0.297 |
| Basal vs Luminal A | 1.48 (0.75-2.89) | 0.256 |
| StageⅢ vsⅠ-Ⅱ | 3.48 (2.03-5.98) | < 0.001 |
