# Supplementary material for: Age‐Associated Four‐Gene Prognostic Signature in Breast Cancer
Source: Cancer Rep (Hoboken). 2026 Sep 1;9(9):e70670. doi: 10.1002/cnr2.70670 (PMC13533818; doi:10.1002/cnr2.70670)
Supplement: Supplementary file 2 [file CNR2-9-e70670-s002.docx]

**Supporting information 3: Multivariable Cox proportional hazards regression analysis of clinicopathological variables for disease-free survival.**

| Variable | HR (95% CI) | P value |
| --- | --- | --- |
| Age group: Young vs Older | 2.25 (1.27-4.00) | 0.006 |
| Histology: ILC vs IDC | 1.04 (0.45-2.42) | 0.921 |
| Luminal B vs Luminal A | 0.78 (0.36-1.69) | 0.534 |
| Her2 vs Luminal A | 1.65 (0.65-4.17) | 0.289 |
| Basal vs Luminal A | 1.51 (0.74-3.09) | 0.261 |
| StageⅢ vsⅠ-Ⅱ | 4.45 (2.41-8.22) | < 0.001 |
