# Supplementary material for: Age‐Associated Four‐Gene Prognostic Signature in Breast Cancer
Source: Cancer Rep (Hoboken). 2026 Sep 1;9(9):e70670. doi: 10.1002/cnr2.70670 (PMC13533818; doi:10.1002/cnr2.70670)
Supplement: Supplementary file 3 [file CNR2-9-e70670-s003.docx]

Supporting information 7: Time-varying coefficient sensitivity analysis in the METABRIC cohort

| **Covariate** | **HR** | **95% CI** | **P value** |
| --- | --- | --- | --- |
| Young age, main effect | 3.26 | 1.283–8.284 | 0.013 |
| Young age × log(time + 1) | 0.787 | 0.611–1.015 | 0.065 |
| Sig4-high vs Sig4-low | 1.337 | 1.113–1.607 | 0.002 |
| ILC vs IDC | 0.991 | 0.694–1.414 | 0.96 |
