# Supplementary material for: Age‐Associated Four‐Gene Prognostic Signature in Breast Cancer
Source: Cancer Rep (Hoboken). 2026 Sep 1;9(9):e70670. doi: 10.1002/cnr2.70670 (PMC13533818; doi:10.1002/cnr2.70670)
Supplement: Supplementary file 4 [file CNR2-9-e70670-s004.docx]

**Supporting information 4: Multivariable Cox proportional hazards regression analysis including the Sig4 score for disease-free survival.**

| Variable | HR (95% CI) | P value |
| --- | --- | --- |
| Age group: Young vs Older | 1.50 (0.83-2.70) | 0.176 |
| Histology: ILC vs IDC | 1.27 (0.55-2.96) | 0.575 |
| Luminal B vs Luminal A | 0.73 (0.34-1.56) | 0.412 |
| Her2 vs Luminal A | 1.24 (0.49-3.16) | 0.651 |
| Basal vs Luminal A | 0.44 (0.18-1.07) | 0.071 |
| StageⅢ vsⅠ-Ⅱ | 3.97 (2.27-6.94) | < 0.001 |
| Sig4 (per 1 SD increase) | 2.45 (1.75-3.44) | < 0.001 |
