# Supplementary material for: Age‐Associated Four‐Gene Prognostic Signature in Breast Cancer
Source: Cancer Rep (Hoboken). 2026 Sep 1;9(9):e70670. doi: 10.1002/cnr2.70670 (PMC13533818; doi:10.1002/cnr2.70670)
Supplement: Supplementary file 5 [file CNR2-9-e70670-s005.docx]

**Supporting information 1: Cox regression coefficients used to calculate the Sig4 score.**

| Gene | β (Cox coefficient) | HR | P value |
| --- | --- | --- | --- |
| C4orf14 | 0.685 | 1.98 | 0.025 |
| LINC01124 | －0.116 | 0.89 | 0.152 |
| ZNF704 | －0.237 | 0.79 | 0.011 |
| AGFG2 | －0.471 | 0.62 | 0.018 |
