# Supplementary material for: Age‐Associated Four‐Gene Prognostic Signature in Breast Cancer
Source: Cancer Rep (Hoboken). 2026 Sep 1;9(9):e70670. doi: 10.1002/cnr2.70670 (PMC13533818; doi:10.1002/cnr2.70670)
Supplement: Supplementary file 6 [file CNR2-9-e70670-s006.docx]

**Supporting information 5: Multivariable Cox proportional hazards analysis for disease-free survival in the young cohort.**

| Variable | HR (95% CI) | P value |
| --- | --- | --- |
| StageⅢ vsⅠ-Ⅱ | 4.27 (1.61-11.29) | 0.003 |
| Non-luminal vs luminal | 0.72 (0.21-2.50) | 0.602 |
| Histology: ILC vs IDC | 3.23 (0.38-27.37) | 0.282 |
| Sig4 (per 1 SD increase) | 2.23 (1.23-4.02) | 0.008 |
