# Supplementary material for: Age‐Associated Four‐Gene Prognostic Signature in Breast Cancer
Source: Cancer Rep (Hoboken). 2026 Sep 1;9(9):e70670. doi: 10.1002/cnr2.70670 (PMC13533818; doi:10.1002/cnr2.70670)
Supplement: Supplementary file 7 [file CNR2-9-e70670-s007.docx]

Supporting information 6: Proportional hazards assumption testing for the reported Cox regression models

| **Cohort** | **Model** | **Covariate** | **Schoenfeld residual test P value** | **Global P value** | **Interpretation** |
| --- | --- | --- | --- | --- | --- |
| TCGA | Sequential Model A | Young age | 0.63 | 0.63 | No evidence of non-proportionality |
| TCGA | Sequential Model B | Young age | 0.82 | 0.72 | No evidence of non-proportionality |
|  |  | Stage III | 0.46 |  | No evidence of non-proportionality |
| TCGA | Sequential Model C | Young age | 0.906 | 0.115 | No evidence of non-proportionality |
|  |  | Stage III | 0.456 |  | No evidence of non-proportionality |
|  |  | Non-luminal subtype | 0.035 |  | Possible non-proportionality |
| TCGA | Sequential Model D | Young age | 0.75 | 0.276 | No evidence of non-proportionality |
|  |  | Stage III | 0.487 |  | No evidence of non-proportionality |
|  |  | Non-luminal subtype | 0.059 |  | No statistically significant non-proportionality |
|  |  | Sig4 per 1-SD increase | 0.196 |  | No evidence of non-proportionality |
| TCGA | Young-subgroup multivariable model | Stage III | 0.93 | 0.46 | No evidence of non-proportionality |
|  |  | Non-luminal subtype | 0.37 |  | No evidence of non-proportionality |
|  |  | ILC histology | 0.28 |  | No evidence of non-proportionality |
|  |  | Sig4 per 1-SD increase | 0.84 |  | No evidence of non-proportionality |
| TCGA | PAM50-adjusted multivariable model | Young age | 0.67 | 0.57 | No evidence of non-proportionality |
|  |  | ILC histology | 0.96 |  | No evidence of non-proportionality |
|  |  | PAM50 subtype | 0.25 |  | No evidence of non-proportionality |
|  |  | Stage III | 0.56 |  | No evidence of non-proportionality |
|  |  | Sig4 per 1-SD increase | 0.2 |  | No evidence of non-proportionality |
| TCGA | Age-cutoff sensitivity model: ≤35 years | Age ≤35 years | 0.943 | 0.416 | No evidence of non-proportionality |
|  |  | Stage III | 0.53 |  | No evidence of non-proportionality |
|  |  | Non-luminal subtype | 0.068 |  | No statistically significant non-proportionality |
|  |  | Sig4 per 1-SD increase | 0.216 |  | No evidence of non-proportionality |
| TCGA | Age-cutoff sensitivity model: ≤40 years | Age ≤40 years | 0.58 | 0.23 | No evidence of non-proportionality |
|  |  | Stage III | 0.44 |  | No evidence of non-proportionality |
|  |  | Non-luminal subtype | 0.06 |  | No statistically significant non-proportionality |
|  |  | Sig4 per 1-SD increase | 0.19 |  | No evidence of non-proportionality |
| TCGA | Age-cutoff sensitivity model: ≤45 years | Age ≤45 years | 0.75 | 0.276 | No evidence of non-proportionality |
|  |  | Stage III | 0.487 |  | No evidence of non-proportionality |
|  |  | Non-luminal subtype | 0.059 |  | No statistically significant non-proportionality |
|  |  | Sig4 per 1-SD increase | 0.196 |  | No evidence of non-proportionality |
| TCGA | Age-cutoff sensitivity model: ≤50 years | Age ≤50 years | 0.358 | 0.221 | No evidence of non-proportionality |
|  |  | Stage III | 0.537 |  | No evidence of non-proportionality |
|  |  | Non-luminal subtype | 0.067 |  | No statistically significant non-proportionality |
|  |  | Sig4 per 1-SD increase | 0.217 |  | No evidence of non-proportionality |
| METABRIC | Young age, univariable | Young age | 0.0011 | 0.0011 | Evidence of non-proportionality |
| METABRIC | Sig4 continuous, univariable | Sig4 per 1-SD increase | 0.097 | 0.097 | No statistically significant non-proportionality |
| METABRIC | Sig4 group, univariable | Sig4-high vs Sig4-low | 0.81 | 0.81 | No evidence of non-proportionality |
| METABRIC | Original multivariable model | Young age | 0.0016 | 8.0 × 10⁻⁹ | Evidence of non-proportionality |
|  |  | Sig4-high vs Sig4-low | 0.7038 |  | No evidence of non-proportionality |
|  |  | Stage III | 0.0098 |  | Evidence of non-proportionality |
|  |  | Non-luminal subtype | 1.8 × 10⁻⁹ |  | Strong evidence of non-proportionality |
|  |  | ILC histology | 0.4592 |  | No evidence of non-proportionality |
| METABRIC | Young-subgroup model | Sig4 per 1-SD increase | 0.053 | 0.053 | Borderline, but not statistically significant |
| METABRIC | Stratified sensitivity model¹ | Young age | 0.039 | 0.128 | Residual evidence of non-proportionality |
|  |  | Sig4-high vs Sig4-low | 0.651 |  | No evidence of non-proportionality |
|  |  | ILC histology | 0.311 |  | No evidence of non-proportionality |

¹ Stage and luminal versus non-luminal subtype were used as stratification factors.
